# Glioblastoma patients’ survival and its relevant risk factors during the pre-COVID-19 and post-COVID-19 pandemic: real-world cohort study in the USA and China

**DOI:** 10.1097/JS9.0000000000001224

**Published:** 2024-02-19

**Authors:** Ling Qin, Haoyi Li, Dao Zheng, Song Lin, Xiaohui Ren

**Affiliations:** a Department of Infectious Diseases, Peking Union Medical College Hospital, Chinese Academy of Medical Science and Peking Union Medical College; b Department of Neurosurgery, Beijing Tiantan Hospital, Capital Medical University, Beijing, China

**Keywords:** COVID-19, diagnosis, glioblastoma, prognosis, risk factors

## Abstract

**Background:**

Although the COVID-19 pandemic has exerted potential impact on patients with glioblastomas (GBMs), it remains unclear whether the survival and its related risk factors of GBM patients would be altered or not during the period spanning from pre-COVID-19 to post-COVID-19 pandemic era. This study aimed to clarify the important issues above.

**Methods:**

Two observational cohorts were utilized, including the nationwide American cohort from the Surveillance, Epidemiology, and End-Results (SEER) and the Chinese glioblastoma cohort (CGC) at our institution during 2018–2020. Demographics, tumour features, treatment regimens and clinical outcomes were collected. Cox regression model, competing risk model, and subgroup and sensitivity analysis were used to dynamically estimate the survival and its relevant risk factors over different diagnosis years from the pre-COVID-19 (2018 and 2019) to post-COVID-19 (2020) pandemic. Causal mediation analysis was further adopted to explore the potential relationship between risk factors and mortality.

**Results:**

This study included 11321 GBM cases in SEER and 226 GBM patients in CGC, respectively. Instead of the diagnostic years of 2018–2020, the prognostic risk factors, such as advanced age, bilateral tumour and absence of comprehensive therapy (surgery combined with chemoradiotherapy), were identified to persistently affect GBM survival independently during the period from 2018 to 2020 in the SEER cohort (all *P* < 0.05). In CGC, lack of comprehensive therapy for GBM patients were restated as survival risk factors during the same timeframe. Causal mediation analysis showed that the effect of comprehensive therapy on all-cause mortality played a determinant role (direct effect value −0.227, 95% CI −0.248 to −0.207), which was partially mediated by age (9.11%) rather than tumour laterality.

**Conclusions:**

As the timeframe shifted from pre-COVID-19 to post-COVID-19 pandemic, survival of GBM patients remained stable, yet advanced age, bilateral tumours, and passive treatment continuingly impacted GBM survival. It is necessary to optimize the comprehensive treatment for GBM patients even in the post-pandemic era.

## Introduction

Highlights

- There were no obvious differences in glioblastoma (GBM) survival before and after the pandemic.
- Advanced age, bilateral tumours, and passive treatment continued to influence GBM survival in the post-pandemic.
- Comprehensive therapy played a determinant role in prolonging GBM survival.

The emerging COVID-19, caused by SARS-CoV2, began spreading rapidly throughout the world at the end of 2019. At present, the transmission of COVID-19 continues to be escalated, posing a serious threat to public health. Zoonotic diseases play a role in facilitating the global spread of COVID-19 during the pandemic^1–4^. Concurrently, regional wars and conflicts not only result in the mass migration of virus carriers, but also contribute to lower vaccination coverage^5^. Due to the limited understanding of the COVID-19 transmission and infectivity up to now, there are still no effective mitigation measures^6^. Despite of considerable efforts in vaccine development^7–10^, prevention^11–14^, and advancements in digital health^15,16^, the COVID-19 pandemic has a significant impact on healthcare systems and vulnerable patients, especially those with malignant brain tumours^4,17^. Glioblastoma (GBM) is the most common malignant primary intracranial lesion, accounting for nearly half of all primary malignant central nervous system tumours. Despite of recent advancements in multimodality therapy for GBMs, the overall prognosis remains dismal, with a 5-year survival rate of approximately 5%^18,19^. As recent studies released^20,21^, the risk of mortality in GBM patients may be even higher in the setting of COVID-19 pandemic.

To date, there have been few studies investigating the real-world survival of GBM patients and its related risk factors under the circumstances of the COVID-19 pandemic. One retrospective study published in 2023 from Germany reported delays in diagnosis and treatment during the pandemic, which led to larger tumour sizes. However, the larger tumour volumes did not result in worse survival outcomes^22^. Another retrospective cohort study released in 2023 from Canada showed that there were no significant delays in time to assessment, time to treatment, or administration of adjuvant therapy during the pandemic compared to the pre-COVID-19 pandemic period, and no difference in survival outcomes as well^23^. These studies were limited by a single research centre, small sample size or focused on special subgroups of GBM, leading to controversial results. Therefore, it is urgent to gather large-scale nationwide surveillance data and multiple cohorts evidence from different countries to thoroughly understand the clinical prognosis of GBM patients and any potential changes in relevant risk factors before and during the COVID-19 pandemic.

Based on the hypothesis that GBM patients diagnosed during the COVID-19 pandemic might face worse clinical outcomes, compared to those diagnosed before the pandemic, we aimed to investigate the survival status and its related risk factors among GBM patients during the period spanning from the pre-COVID-19 and post-COVID-19 pandemic. We leveraged a large nationwide cohort database in the United States and a Chinese glioblastoma cohort (CGC) from one of the largest brain tumour research centres in China to access this concern.

## Methods

### Study design, data source and patient assessment

This study was a retrospective observational cohort study that adhered to the STROCSS criteria^24^, Supplemental Digital Content 1, http://links.lww.com/JS9/B957. The targeted population of this study originated from the American nationwide cohort based on the Surveillance, Epidemiology, and End-Results (SEER) 22 registries (2018–2020) in the United States, and the representative CGC at our institution in China. This study was approved by IRB of our institution (KY2021-115-02), and the informed consent from patients was waived.

**Figure 1:**
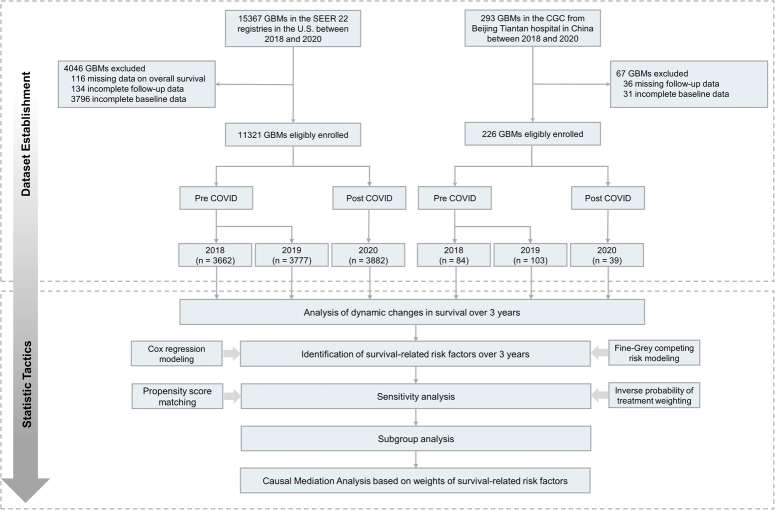
Flowchart of dataset establishment and statistic tactics. CGC, Chinese glioblastoma cohort; GBM, glioblastoma; SEER, Surveillance, Epidemiology, and End-Results.

SEER*Stat (v8.4.2) was used to inquiry about all cases (*n* = 15367) with histopathologically confirmed diagnosis of GBM (International Classification of Diseases for Oncology-Third Edition [ICD-O-3] codes 9440, 9441, 9442, 9445) between 2018 and 2020. A detailed description of the search process was provided in Supplementary Figure 1, Supplemental Digital Content 2, http://links.lww.com/JS9/B958. The CGC database was searched for patients (*n* = 293) who were surgically treated and pathologically diagnosed GBM using the 2016 WHO classification schemes from January 2018 to December 2020. Patients with missing data on overall survival, or incomplete follow-up or baseline data were excluded (study flowchart in Fig. 1).

### Covariable collection

Within the SEER cohort, demographic information was collected, including age, sex and race. Socioeconomic data were recorded, such as median house income codes calculated from country-level data from the American Community Survey 5-year estimates and rural/urban continuum codes calculated from the United States Department of Agriculture classification codes. Tumour-specific parameters were also collected, including the year of diagnosis, histological type (ICD-O-3 9440/3, 9441/3, 9442/3 9445/3), total number of in situ/malignant tumour, primary lesion, tumour site (c71.0–c72.0, c72.3, c72.5, c75.1, c75.3) and laterality. Therapeutic information was documented, including surgery (2006+), radiotherapy (2003+) and chemotherapy (2004+). Treatment delay was defined as the time in months from diagnosis to treatment. The approach to covariate collection above was similarly adopted in the CGC.

### Clinical outcomes and COVID-19 pandemic exposure

Clinical outcomes included all-cause mortality, defined as the time in months from diagnosis to death owing to any cause, and tumour-specific mortality defined as the time in months from diagnosis to death due to GBM. To further characterize survival-related risk factors in GBM patients before and during the COVID-19 pandemic, the diagnosis year of GBM was categorized as the pre-COVID-19 pandemic era (diagnosed in 2018 and 2019) and the post-COVID-19 pandemic era (diagnosed in 2020).

### Statistic tactics

Categorical variables were expressed as percentages, while continuous variables were expressed as median with interquartile range (IQR). Descriptive statistics were computed using Pearson’s χ^2^ test, Fisher’s exact test or Kruskal–Willi’s rank sum test, as appropriate. For survival analysis, Cox proportional hazard regression models were used to estimate the hazard ratios (HRs) of clinical outcomes associated with potential risk factors. Covariates included demographic factors, tumour features and treatment regimens. Covariates with a *p* value of less than 0.01 in the univariate Cox regression analysis were identified for inclusion in the multivariable Cox models. The proportional hazard assumption of the Cox model was assessed using Schoenfeld residuals tests (*P* > 0.1 indicating no deviation from the assumption). Multicollinearity was tested using the variance inflation factor (VIF < 10 indicating no statistically significant interaction terms). Additionally, multivariable Fine-Gray competing risk models were calculated using the “cmprsk” R package. Moreover, sensitivity analyses were conducted using the propensity score matching (PSM) method and inverse probability of treatment weighting (IPTW) adjustment. Based on the estimated propensity of each patient, a 1:1 matched analysis for each identified risk factor was performed using the nearest-neighbour matching method without replacement and with a caliper radius of 0.1. Moreover, adjustment for all covariates was performed through the IPTW methodology using the previously similar Cox regression analysis. Estimates were presented as HRs with 95% CIs. To display the clinical survival, unadjusted and all-cause adjusted Kaplan–Meier curves were plotted. In order to investigate the survival-related determinants among GBM patients in different subgroups, we further employed Kaplan–Meier plots to conduct a stratified analysis by latent class of different risk factors. In addition, the restricted cubic spine (RCS) functions developed by the “splines” R package were employed to address the potential nonlinearity of the dose-response correlations between age and clinical outcomes. Finally, considering the potential interaction between the different survival-related risk factors, we conducted causal mediation analysis to access the plausibility of the causal assumption between the identified factors and survival using the SPSSAU. (Version 23.0). Bias-corrected bootstrap with 1000 replications was used to estimate average mediation effects. Statistical computations were performed using software programs (SPSS v 26.0 IBM and R v 4.2.2). A *p* value less than 0.05 for two-tailed tests was considered statistically significant.

## Results

### Study population

A total of 11321 eligible cases from SEER cohort were enroled in this analysis after careful screening (Fig. 1). Among them, 3662 (32.3%) and 3777 (33.4%) individuals were diagnosed with GBM in 2018 and 2019, respectively, while 3882 (34.3%) were diagnosed in 2020. There were significant differences in median household income, tumour laterality, treatment delay, radiotherapy, follow-up time and mortality (all with *P* < 0.05) among the three groups. Moreover, a Chinese cohort from CGC, consisting of 226 eligible patients was included between the pre-COVID and post-COVID pandemic periods. Among them, 84 (37.2%) and 103 (45.6%) patients were diagnosed with GBM in 2018 and 2019, respectively. Thirty-nine patients (17.2%) were diagnosed in 2020. Baseline differences were minimal among the three groups, except for radiotherapy and chemotherapy (Table 1).

**Table 1:** Baseline characteristics between the pre-COVID-19 and post-COVID-19 pandemic in SEER and CGC databases.

SEER					CGC				
	Pre COVID, *n* (%)		Post COVID, *n* (%)			Pre COVID, *n* (%)		Post COVID, *n* (%)	
Variables	2018 (*n* = 3662)	2019 (*n* = 3777)	2020 (*n* = 3882)	*p*	Variables	2018 (*n* = 84)	2019 (*n* = 103)	2020 (*n* = 39)	*p*
Demographics					Demographics				
Age				0.574	Age				0.743
< 65 years	1887 (51.5)	1909 (50.5)	1957 (50.4)		< 65 years	75 (89.3)	90 (87.4)	33 (84.6)	
≥ 65 years	1775 (48.5)	1868 (49.5)	1925 (49.6)		≥ 65 years	9 (10.7)	13 (12.6)	6 (15.4)	
Sex				0.919	sex				0.638
Female	1488 (40.6)	1530 (40.5)	1560 (40.2)		Female	31 (36.9)	35 (34.0)	11 (28.2)	
Male	2174 (59.4)	2247 (59.5)	2322 (59.8)		Male	53 (63.1)	68 (66.0)	28 (71.8)	
Race				0.148	Race				> 0.999
Hispanic	535 (14.6)	612 (16.2)	587 (15.1)		Han	84 (100.0)	102 (99.0)	39 (100.0)	
Non-Hispanic	3127 (85.4)	3165 (83.8)	3295 (84.9)		Non-Han	0 (0.0)	1 (1.0)	0 (0.0)	
Median household income				< **0.001**	Residency				0.127
< $ 75 000	2046 (55.9)	1630 (43.2)	1670 (43.0)		Rural	36 (42.9)	59 (57.3)	18 (46.2)	
≥ $ 75 000	1616 (44.1)	2147 (56.8)	2212 (57.0)		Urban	48 (57.1)	44 (42.7)	21 (53.8)	
Rural/Urban Continuum				0.347					—
< 1 million population	1053 (28.8)	1035 (27.4)	1048 (27.0)			—	—	—	
> 1 million population	2203 (60.2)	2303 (61.0)	2361 (60.8)			—	—	—	
Unknown	406 (11.0)	439 (11.6)	473 (12.2)			—	—	—	
Tumour features					Tumour features				
Tumour site				0.602	Tumour site				0.869
Supratentorial	2791 (76.2)	2874 (76.1)	2961 (76.3)		Supratentorial	73 (86.9)	91 (88.3)	34 (87.2)	
Non-supratentorial	511 (14.0)	510 (13.5)	555 (14.3)		Non-supratentorial	1 (1.2)	3 (3.0)	1 (2.5)	
Unknown	360 (9.8)	393 (10.4)	366 (9.4)		Unknown	10 (11.9)	9 (8.7)	4 (10.3)	
Laterality				**0.004**	Laterality				0.310
Non-bilateral	3287 (89.8)	3419 (90.5)	3425 (88.2)		Non-bilateral	39 (46.4)	44 (42.7)	20 (51.3)	
Bilateral	75 (2.0)	52 (1.4)	73 (1.9)		Bilateral	35 (41.7)	54 (52.4)	17 (43.6)	
Unknown	300 (8.2)	306 (8.1)	384 (9.9)		Unknown	10 (11.9)	5 (4.9)	2 (5.1)	
Treatment delay				< **0.001**	No. in situ/malignant tumours				0.179
= 0	2799 (76.4)	2933 (77.7)	3121 (80.4)		= 1	68 (81.0)	93 (90.3)	34 (87.2)	
> 0	863 (23.6)	844 (22.3)	761 (19.6)		> 1	16 (19.0)	10 (9.7)	5 (12.8)	
No. *in situ*/malignant tumours				0.493	Primary lesion				0.179
= 1	2959 (80.8)	3090 (81.8)	3144 (81.0)		Yes	68 (81.0)	93 (90.3)	34 (87.2)	
> 1	703 (19.2)	687 (18.2)	738 (19.0)		No	16 (19.0)	10 (9.7)	5 (12.8)	
Primary lesion				0.566	Histological type				0.594
Yes	3036 (82.9)	3159 (83.6)	3214 (82.8)		GBM subtype	81 (96.4)	101 (98.1)	39 (100.0)	
No	626 (17.1)	618 (16.4)	668 (17.2)		Non-GBM subtype	3 (3.6)	2 (1.9)	0 (0.0)	
Histological type				0.186					
GBM subtype	3534 (96.5)	3672 (97.2)	3766 (97.0)						
Non-GBM subtype	128 (3.5)	105 (2.8)	116 (3.0)						
Treatment					Treatment				
Surgical treatment					Surgical treatment				0.179
Surgery	3224 (88.0)	3391 (89.8)	3509 (90.4)		Surgery	68 (81.0)	93 (90.3)	34 (87.2)	
No surgery	434 (11.9)	383 (10.1)	372 (9.6)		No surgery	16 (19.0)	10 (9.7)	5 (12.8)	
Unknown	4 (0.1)	3 (0.1)	1 (0.0)		Unknown				
Radiotherapy				**0.013**	Radiotherapy				**0.018**
Yes	2343 (64.0)	2326 (61.6)	2360 (60.8)		Yes	65 (77.4)	81 (78.6)	38 (97.4)	
No/Unknown	1319 (36.0)	1451 (38.4)	1522 (39.2)		No/Unknown	19 (22.6)	22 (21.4)	1 (2.6)	
Chemotherapy				0.107	Chemotherapy				**0.032**
Yes	2690 (73.5)	2697 (71.4)	2785 (71.7)		Yes	68 (81.0)	82 (79.6)	38 (97.4)	
No/unknown	972 (26.5)	1080 (28.6)	1097 (28.3)		No/Unknown	16 (19.0)	21 (20.4)	1 (2.6)	
Comprehensive therapy^a^				0.328	Comprehensive therapy^a^				0.078
Yes	1868 (51.0)	1871 (49.5)	1921 (49.5)		Yes	63 (75.0)	80 (77.7)	36 (92.3)	
No/Unknown	1794 (49.0)	1906 (50.5)	1961 (50.5)		No	21 (25.0)	23 (22.3)	3 (7.7)	
Clinical outcomes					Clinical outcomes				
Follow-up, month, median (IQR)	11 (4–20)	10 (4–15)	4 (1–7)	< **0.001**	Follow-up, month, median (IQR)	16 (11–23)	17 (11–30)	20 (13–24)	0.392
All-cause mortality				< **0.001**	All-cause mortality				0.351
Yes	629 (17.2)	1259 (33.3)	2723 (70.1)		Yes	23 (27.4)	20 (19.4)	11 (28.2)	
No	3033 (82.8)	2518 (66.7)	1159 (29.9)		No	61 (72.6)	83 (80.6)	28 (71.8)	
Tumour-specific mortality				< **0.001**	Tumour-specific mortality				0.375
Yes	806 (22.0)	1406 (37.2)	2835 (73.1)		Yes	24 (28.6)	21 (20.4)	11 (28.2)	
No	2796 (76.4)	2329 (61.7)	1030 (26.5)		No	60 (71.4)	82 (79.6)	28 (71.8)	
Unknown	60 (1.6)	42 (1.1)	17 (0.4)		Unknown	—	—	—	
Other cause of mortality				< **0.001**	Other cause of mortality				0.544
Yes	3425 (93.6)	3588 (95.0)	3753 (96.7)		Yes	83 (98.8)	103 (100.0)	39 (100.0)	
No	177 (4.8)	147 (3.9)	112 (2.9)		No	1 (1.2)	0 (0.0)	0 (0.0)	
Unknown	60 (1.6)	42 (1.1)	17 (0.4)		Unknown	—	—	—	

Boldface type indicates statistical significance with two-sided *P* < 0.05.

CGC, Chinese glioblastoma cohort; GBM, glioblastoma; IQR, interquartile range; n, number; SEER, Surveillance, Epidemiology, and End-Results.

a Comprehensive therapy includes surgery combined with chemoradiotherapy.

### GBM survival during the pre-COVID-19 and post-COVID-19 Pandemic

**Figure 2:**
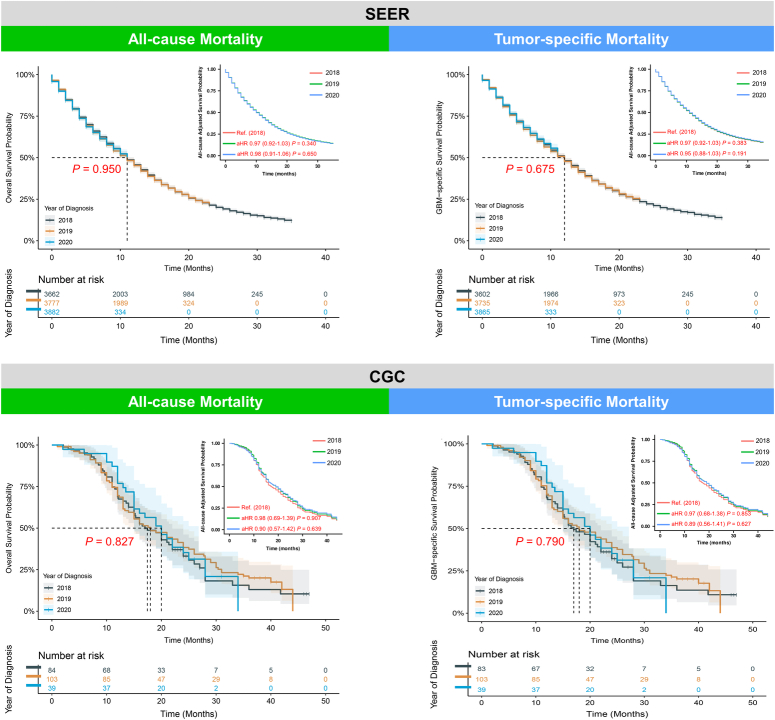
Uni and adjusted Kaplan–Meier curves stratified by diagnostic year for GBM patients in both SEER cohort and CGC. In SEER cohort, all-cause adjusted Kaplan–Meier curves were plotted after control of all covariates, including age, sex, race, median household income, rural/urban continuum, tumour site, laterality, treatment delay, no. of situ/malignant tumours, primary lesion, histological type, surgical treatment, radiotherapy and chemotherapy. In CGC, survival curves were plotted after adjusting for age, sex, race, residency, tumour site, laterality, no. of situ/malignant tumours, primary lesion, histological type, surgical treatment, radiotherapy and chemotherapy. aHR, adjusted hazard ratio; CGC, Chinese glioblastoma cohort; SEER, Surveillance, Epidemiology, and End-Results.

In the SEER cohort, Kaplan–Meier curves showed no significant differences in the median all-cause (*P* = 0.950) and tumour-specific mortalities (*P* = 0.675) among different diagnosis years ranging from 2018 to 2020. After adjusting for all covariates, the median survival time of GBM showed no significant difference in 2019 and 2020 compared to the year of 2018 (Fig. 2). Similar results were observed in the CGC (Fig. 2). Furthermore, there were no significant associations between all-cause mortality and the year of diagnosis in Cox regression modelling in both the SEER database and CGC. So did tumour-specific mortality.

### Identification of risk factors related to survival during the pre-COVID-19 and post-COVID-19 pandemic

**Figure 3:**
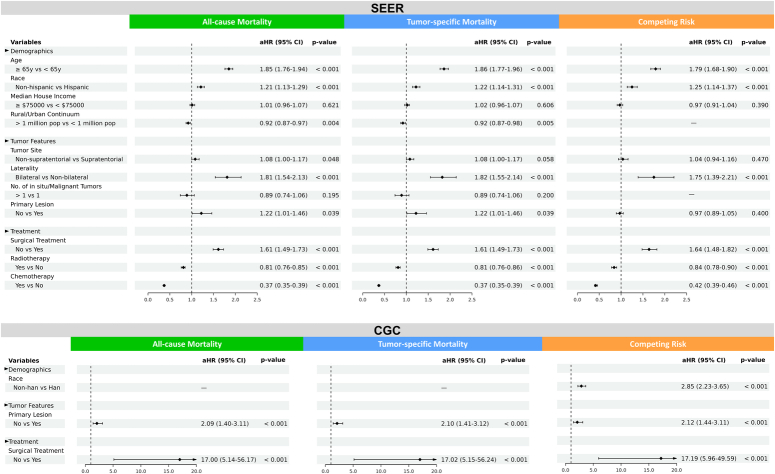
Multivariable analysis of prognostic risk factors in both SEER cohort and CGC. Covariables with a *p* value less than 0.01 in the univariate survival analysis (Cox regression or Competing risk model) were shown in this figure and added to the multivariable survival models to further identify independent survival-related risk factors. aHR, adjusted hazard ratio; CGC, Chinese glioblastoma cohort; pop, population; SEER, Surveillance, Epidemiology, and End-Results; y, year (s).

In the SEER cohort, multivariable Cox models were conducted to assess the correlations between potential risk factors and all-cause mortality during the period (2018–2020) among GBM patients. Worse outcomes were significantly related to advanced age [adjusted hazard ratio (aHR), 1.85 (95% CI, 1.76–1.94), *P* < 0.001], non-Hispanic race [aHR, 1.21 (95% CI, 1.13–1.29), *P* < 0.001], less 1 million population in rural/urban continuum [aHR, 0.92 (95% CI, 0.87–0.97), *P* = 0.004], non-supratentorial tumour [aHR, 1.08 (95% CI, 1.00–1.17), *P* = 0.048], bilateral lesion [aHR, 1.81 (95% CI, 1.54–2.13), *P* < 0.001], non-primary tumour [aHR, 1.22 (95% CI, 1.01–1.46), *P* = 0.039], no surgery [aHR, 1.61 (95% CI, 1.49–1.73), *P* < 0.001], no radiotherapy [aHR, 0.81 (95% CI, 0.76–0.85), *P* < 0.001] and no chemotherapy [aHR, 0.37 (95% CI, 0.35–0.39), *P* < 0.001]. Similar findings were observed for the risk factors of tumour-specific mortality in GBM patients (Fig. 3). These results were further confirmed by competing risk models (Fig. 3). Moreover, in order to further elucidate the effect of risk factors on survival, multivariable Cox analysis were conducted in each calendar year from 2018 to 2020. The results demonstrated that older age, bilateral lesion, and lack of surgery, radiotherapy or chemotherapy were continuingly associated with unfavourable survival regardless of the diagnostic year. (Supplementary Tables 1-3, Supplemental Digital Content 3, http://links.lww.com/JS9/B959, Supplemental Digital Content 4, http://links.lww.com/JS9/B960, Supplemental Digital Content 5, http://links.lww.com/JS9/B961).

For the CGC, multivariable Cox models, based on covariate adjustment, showed that patients without surgical treatment or primary lesion experienced a higher risk of all-cause and tumour-specific mortalities before and during the COVID-19 pandemic (Fig. 3). Additionally, competing risk model identified non-Han race as a significant risk factor related to worse survival (Fig. 3). Furthermore, non-primary lesion and refusal of surgery were considered as fixed risk factors significantly related to mortality during the timeframe ranging from 2018 to 2020 (Supplementary Tables 4-6, Supplemental Digital Content 6, http://links.lww.com/JS9/B962, Supplemental Digital Content 7, http://links.lww.com/JS9/B963, Supplemental Digital Content 8, http://links.lww.com/JS9/B964).

A sensitivity analysis of fixed risk factors related to survival over the period of 2018–2020 was presented in Supplementary Table 7, Supplemental Digital Content 9, http://links.lww.com/JS9/B965. After PSM and IPTW adjustment, the multivariable Cox model revealed significant associations between poor outcomes and fixed risk factors, including older age, bilateral tumour, and passive treatment strategies (refusal of surgery, radiotherapy and/or chemotherapy) in the SEER cohort. Furthermore, IPTW-adjusted Cox models showed significant associations between non-primary lesion, lack of surgery and high mortality in the CGC.

### Subgroup analysis of survival-related risk factors in the SEER cohort

**Figure 4:**
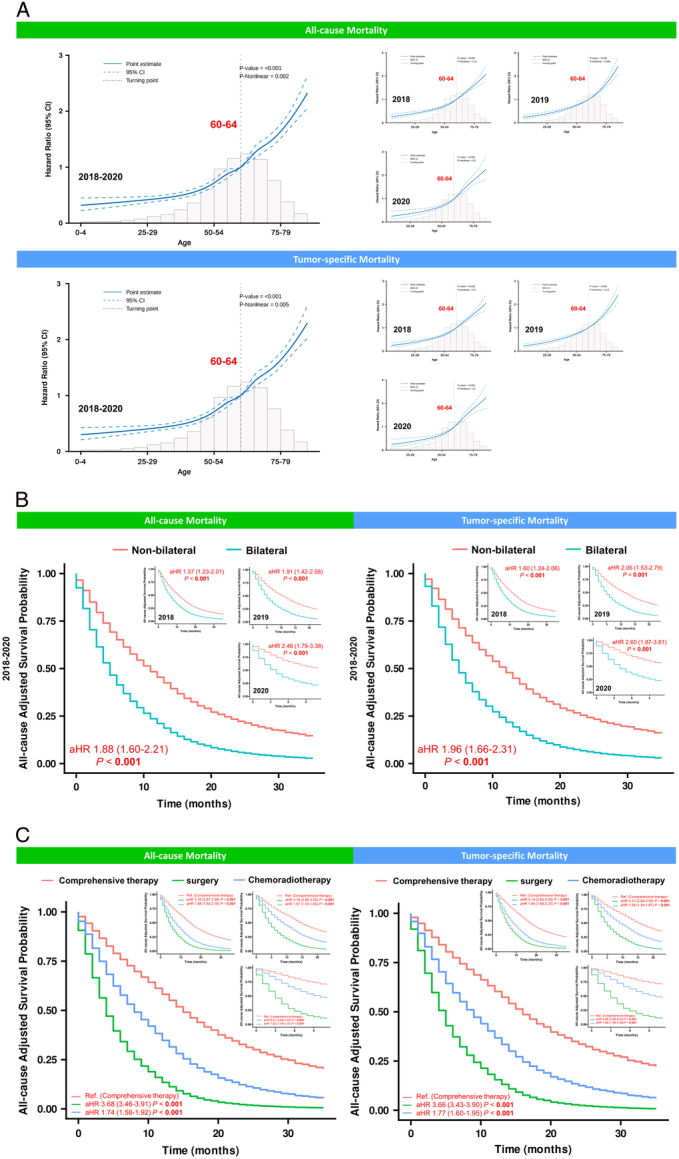
Subgroup analysis of survival-related risk factors in the SEER cohort. (A) Restricted cubic spline (RCS) plots of relationships between age and clinical outcomes during the period from 2018 to 2020. RCS plots of age during the whole study timeframe was performed after adjusting for year of diagnosis, gender, race, median household income, rural/urban continuum, tumour site, laterality, treatment delay, no. of situ/malignant tumours, primary lesion, histological type, surgical treatment, radiotherapy and chemotherapy. RCS plots of age in each year of study timeframe was performed after adjusting for sex, race, median household income, rural/urban continuum, tumour site, laterality, treatment delay, no. of situ/malignant tumours, primary lesion, histological type, surgical treatment, radiotherapy and chemotherapy. (B) All-cause Kaplan–Meier curves on association between the tumour laterality and clinical outcomes during the period from 2018 to 2020. The survival curves stratified by tumour laterality during the whole study timeframe were plotted after adjusting for year of diagnosis, age, sex, race, median household income, rural/urban continuum, tumour site, treatment delay, no. of situ/malignant tumours, primary lesion, histological type, surgical treatment, radiotherapy and chemotherapy. The survival curves stratified by tumour laterality in each year of the study timeframe were plotted after adjusting for age, sex, race, median household income, rural/urban continuum, tumour site, treatment delay, no. of situ/malignant tumours, primary lesion, histological type, surgical treatment, radiotherapy and chemotherapy. (C) All-cause adjusted Kaplan–Meier curves on association between the treatment regimens and clinical outcomes during the period from 2018 to 2020. The survival curves stratified by treatment regimens during the whole study timeframe were plotted after adjusting for year of diagnosis, age, sex, race, median household income, rural/urban continuum, tumour site, laterality, treatment delay, no. of situ/malignant tumours, primary lesion and histological type. The survival curves stratified by treatment regimens in each year of the study timeframe were plotted after adjusting for age, sex, race, median household income, rural/urban continuum, tumour site, laterality, treatment delay, no. of situ/malignant tumours, primary lesion and histological type. aHR, adjusted hazard ratio; CGC, Chinese glioblastoma cohort.

Based on the findings above, age, tumour laterality, and treatment regimens were continuingly influencing the clinical outcomes of GBM patients during the period of 2018–2020. Concerning aging as a survival-related risk factor, RCS plots displayed a mirrored L-shaped association between age and all-cause or tumour-specific mortality during the period of 2018–2020 after adjusting for all covariates (all with *P* < 0.001). Further details on RCS plots revealed an inflection point of age over 65 years with dismal prognosis, which was also found in the RCS analysis for the year of 2018, 2019 and 2020, respectively (Fig. 4A). Additionally, GBM patients with bilateral tumours significantly experienced worse survival after adjusting for all covariates over the whole study timeframe, as well as in the year of 2018, 2019 and 2020, respectively (Fig. 4B). With regards to the GBM therapy, the subgroup analysis of the associations between different treatment strategies and survival showed that GBM patients who received either single surgery [aHR, 3.68 (95% CI, 3.46–3.91), *P* < 0.001] or single chemoradiotherapy [aHR, 1.74 (95% CI, 1.58–1.92), *P* < 0.001] had higher all-cause mortality than those who received surgery combined chemoradiotherapy (i.e. comprehensive therapy) during the period of 2018–2020. Similar associations between treatment strategies and tumour-specific mortality were also found (Fig. 4C). Regarding each year of diagnosis in our study, the favourable prognosis of GBM patients, who had received comprehensive therapy rather than surgery or chemoradiotherapy alone, was demonstrated (Fig. 4C).

### Potential causal mediation analysis based on survival-related risk factors in the SEER Cohort

**Figure 5:**
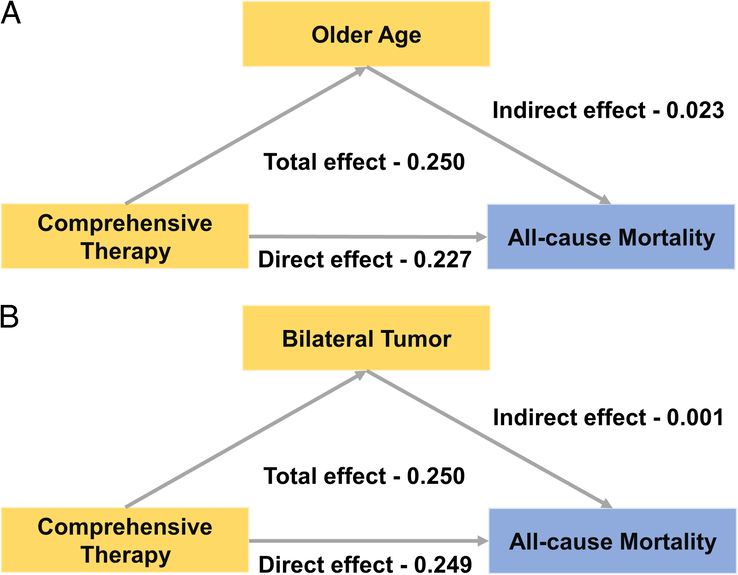
Causal mediation effect path model (A) Causal mediation analysis mediated by age between the comprehensive therapy and all-cause mortality. (B) Causal mediation analysis mediated by tumour laterality between the comprehensive therapy and all-cause mortality. Yellow block represents exposures and mediators. Blue block represents outcomes. Grew arrow represents path direction between the covariates in each model.

There were significantly mutual correlations among fixed survival-related risk factors (age, tumour laterality and treatment regimens) (Supplementary Table 8, Supplemental Digital Content 10, http://links.lww.com/JS9/B966). In order to disclose the potential interactions among the risk factors above and their mechanisms affecting survival, we conducted the potential causal mediation analysis. We hypothesized that the impact of comprehensive therapy on GBM survival might be partially or fully medicated by either age or tumour laterality. In Fig. 5A, there was a significant total effect of comprehensive therapy on all-cause mortality (total effect value −0.250, 95% CI −0.271 to −0.230). After accounting for the mediating effect of age, the direct effect of comprehensive therapy on all-cause mortality remained significant (direct effect value −0.227, 95% CI −0.248 to −0.207) with a proportion of 9.11% mediated by age. (Supplementary Tables 9 and 10, Supplemental Digital Content 11, http://links.lww.com/JS9/B967, Supplemental Digital Content 12, http://links.lww.com/JS9/B968). However, due to the weak mediating effect of tumour laterality (indirect effect value −0.001, 95% CI −0.003 to 0.000), the impact of comprehensive therapy on mortality could not be mediated by tumour laterality (direct effect value −0.249, 95% CI −0.269 to −0.228) (Fig. 5B, Supplementary Tables 11 and 12, Supplemental Digital Content 13, http://links.lww.com/JS9/B969, Supplemental Digital Content 14, http://links.lww.com/JS9/B970).

## Discussion

GBM, considered as one of the most lethal brain tumours, had posed significant challenges despite active and timely treatment. Since the first case with SARS-CoV2 was reported in Wuhan in 2019, various countries had implemented optimal regulations to manage brain tumour patients, particularly vulnerable GBM individuals during the pandemic^25^. To the best of our knowledge, this is the first study with multi-cohorts from different countries worldwide to dynamically investigate clinical survival and its relevant risk factors among GBM patients before and during the COVID-19 pandemic era. Several critical risk factors continuingly impacting survival were identified, including advanced age, bilateral lesion, non-primary tumour, refusal of surgery combined with chemoradiotherapy during the period spanning from the pre-COVID-19 to post-COVID-19 pandemic. In addition, our study highlighted the potential mediating role of aging and the determinant impact of comprehensive therapy on the survival of GBM patients through robust statistical deduction.

As previously speculated, the COVID-19 pandemic may significantly impact the incidence, diagnosis, treatment, and clinical outcomes of cancer worldwide. Early reports from different countries suggested that cancer patients with COVID-19 infection might face an increased risk of severe clinical events, such as admission to the intensive care unit, need for invasive mechanical ventilation, or death^26–28^. A recent meta-analysis has quantitatively concluded that cancer is associated with worse clinical outcomes among individuals with COVID-19^29^. However, our study indicated that neither all-cause mortality nor tumour-specific mortality of GBM patients was significantly related to the diagnostic year spanning from pre-COVID-19 and post-COVID-19 pandemic. Due to the lack of COVID-19 infection records and specific information on prevention and control policies during the COVID-19 pandemic, we were unable to accurately speculate the impact of the pandemic on the prognosis of GBM. However, our results showed that the rate of treatment delay was significantly lower during the pandemic (19.6%) than before the COVID-19 pandemic (2018, 23.6%; 2019, 22.3%) in the SEER cohort. Based on that, we weakly speculated that the unexpected results in our study may be attributed to the timely adjustment of medical resource allocation, even though China or the USA government adopted different precautions against the COVID-19 outbreak.

Aging, as one of the most critical risk factors, significantly affected the survival of GBM patients^30^. Previous studies reported that age-dependent changes in the brain and immune system may lead to poor outcomes in GBM individuals^31–33^. Our findings supported this notion, emphasizing that advanced age was significantly related to poor prognosis in each of the years before and during the COVID-19 pandemic. This study further underscored the vulnerable population threshold at the age of over 65 in the USA, despite of being in the setting of the COVID-19 pandemic. In contrast to the results of the SEER cohort, no significant relationship between age and survival was observed in the Chinese counterparts. This discrepancy can possibly be attributed to the high aptness of Chinese individuals to actively accept comprehensive therapy provided by healthcare givers (~80% of cases), which may minimize the harmful effect of aging on GBM survival. Additionally, a recent study reported that there has been less improvement in survival among elderly GBM patients in the USA in recent years, with a slightly increased mortality by 0.4% per year from 2009 to 2018^34^. As a result, concerns about improving survival in elderly GBM have remained a focus in the post-COVID-19 era.

According to the National Comprehensive Cancer Network guidelines, it was pivotal for survival improvement that GBM patients underwent surgery, radiotherapy and chemotherapy before the COVID-19 pandemic^35^. Our study further highlighted the significant priority of three key interventions on the GBM patients’ survival after entering pandemic. Previous study has summarized that the surgery-based multimodality therapeutic system remained the cornerstone of the GBM treatment^30^. Our subgroup analysis revealed that compared to either surgery or chemoradiotherapy alone, comprehensive therapy for extending the GBM patients’ survival should be prioritized not only in the pre-COVID-19 era but also in the post-pandemic period. In addition, causal mediation analysis suggested a significant contribution of comprehensive therapy to survival improvement in GBM patients after considering the mediating effect of age. Interestingly, we observed that the median survival time in the Chinese cohort was significantly increased compared to that in the USA cohort. One of the possible explanations was that ~50% of GBM patients in the SEER cohort accomplished comprehensive therapy, while nearly 80% of the counterparts in CGC adopted it. Generally, we strongly proposed that comprehensive therapy should still be highlighted for GBM patients’ management in the post-COVID-19 era.

Before the COVID-19 pandemic, previous reports had elucidated the impact of tumour features on GBM prognosis^36^. Our study supported this finding and indicated that GBM patients with bilateral and non-primary characteristics were significantly associated with poor prognosis before and during the pandemic. However, further causal mediation analysis disclosed that the comprehensive therapy should still play a determinant role in the prognosis of GBM patients. Therefore, it is critical to optimize the therapeutic strategy in the future.

### Limitations

Firstly, both cohorts from the USA and China did not involve information on detailed COVID-19 infection records and control and prevention policies as well, which inevitably led to a bias in estimating the direct action of the COVID-19 pandemic on survival. However, the causes of death in two cohorts are mainly tumour-specific rather than COVID-19 itself, which allowed further survival analysis possible. Secondly, there was an obvious sampling bias, given that only patients registered were included in the SEER database. However, GBM patients registered in the SEER database were observed to be benefit from comprehensive therapy. Finally, due to the short post-COVID-19 timeframe in this study, it is essential to investigate the clinical prognosis of GBM patients over a longer time horizon in the post-COVID-19 era.

## Conclusion

Based on the current findings from the SEER and Chinese cohorts, it was thrilling to find that the survival status for GBM patients did not show an obvious difference during the diagnostic year from pre-COVID to post-COVID pandemic. As the study timeframe dynamically shifted from 2018 to 2020, advanced age, bilateral tumour and absence of surgery combined with chemoradiotherapy were identified to contribute to GBM death continuingly. Based on causal mediation analysis, this study further confirmed the determinant role of comprehensive therapy in improving GBM patients’ survival, which was partially mediated by age. It was pivotal to optimize the comprehensive treatment of GBM individuals in the future.

## Ethical approval

This study was approved by IRB of Beijing Tiantan hospital (KY2021-115-02).

## Consent

Due to this study with retrospective design, the informed consent from patients was waived.

## Source of funding

This work was supported by Capital Health Research and Development of Special Beijing (2022-2-1072).

## Author contribution

L.Q.: conceptualization, data curation, formal analysis, investigation, methodology, software, writing—original draft, writing—review and editing. H.L.: conceptualization, data curation, formal analysis, investigation, software, writing—original draft, visualization, writing—review and editing. D.Z.: writing—review and editing. S.L.: supervision, writing—review and editing. X.R.: funding acquisition, supervision, writing—review and editing.

## Conflicts of interest disclosure

The authors declare that they have no competing interests.

## Research registration unique identifying number (UIN)

The study has registered with ClinicalTrials.gov, study number NCT03392545.

## Guarantor

Xiaohui Ren.

## Data availability statement

For SEER cohort, this raw data are open and available for public use. For CGC cohort, the raw data associated with the findings of this study are obtained from the corresponding author on reasonable request after the approval of the local IRB.

## Provenance and peer review

Not applicable.

## Supplementary Material
