# Supplementary material for: Glioblastoma patients’ survival and its relevant risk factors during the pre-COVID-19 and post-COVID-19 pandemic: real-world cohort study in the USA and China
Source: Int J Surg. 2024 Feb 19;110(5):2939–49. doi: 10.1097/JS9.0000000000001224 (PMC11093471; doi:10.1097/JS9.0000000000001224)
Supplement: Supplementary file 2 [file js9-110-2939-s002.docx]

**Supplementary Figure 1.** Search process in SEER

| **STEP 1** database selection | **STEP 2** covariable selection |
| --- | --- |
| 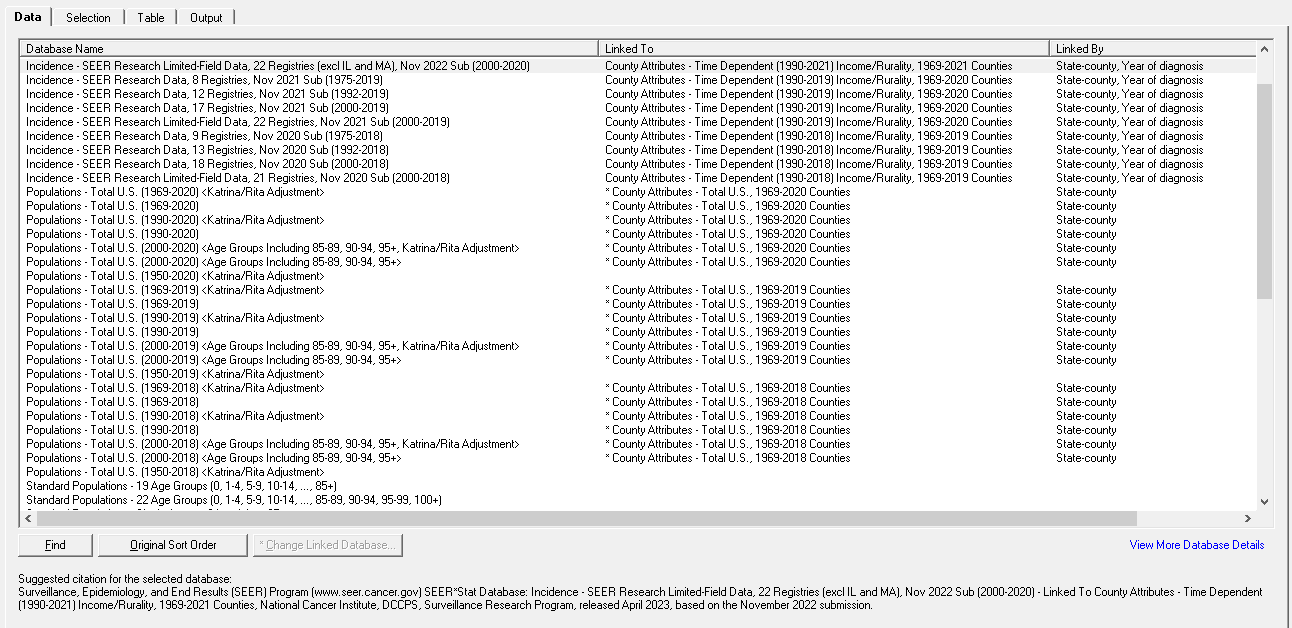 | 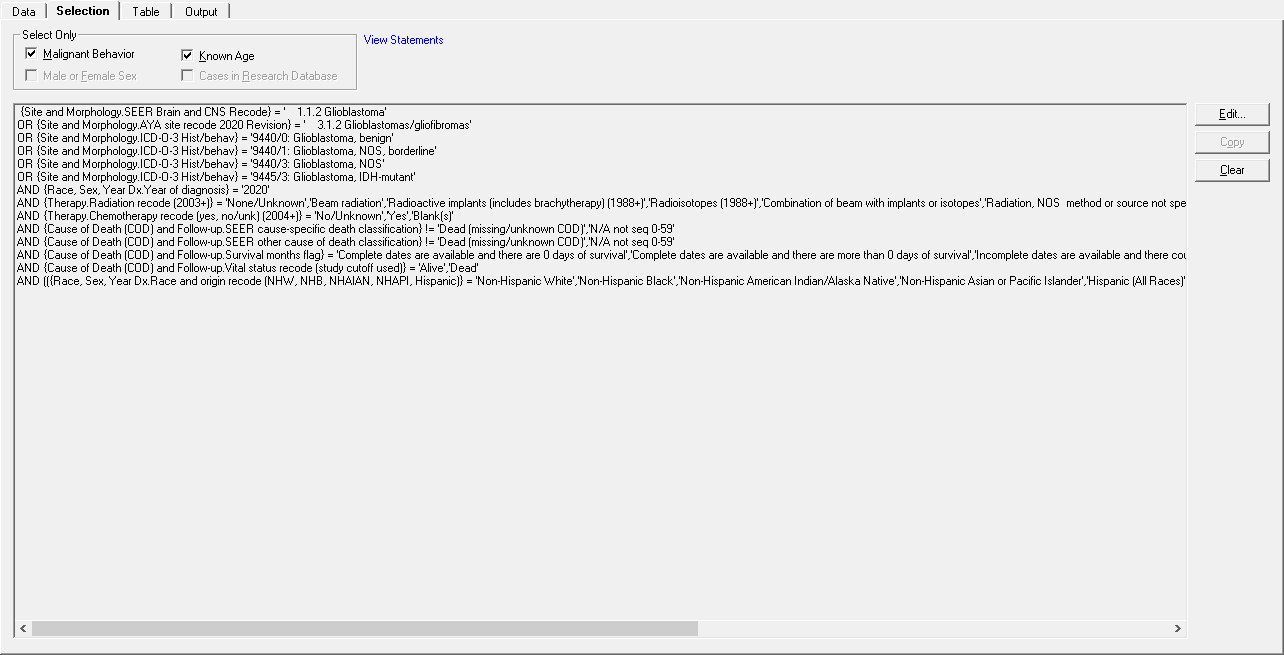 |
| **STEP 3** table development | |
| 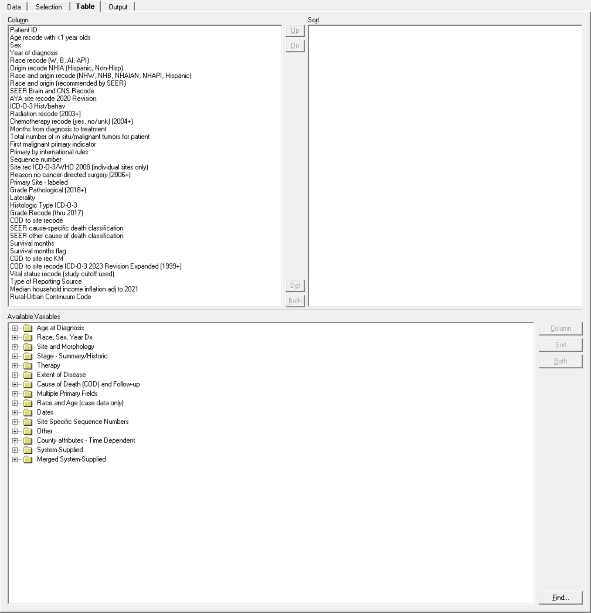 | |

Abbreviation: EER, Surveillance, Epidemiology, and End-Results
