# Supplementary material for: Glioblastoma patients’ survival and its relevant risk factors during the pre-COVID-19 and post-COVID-19 pandemic: real-world cohort study in the USA and China
Source: Int J Surg. 2024 Feb 19;110(5):2939–49. doi: 10.1097/JS9.0000000000001224 (PMC11093471; doi:10.1097/JS9.0000000000001224)
Supplement: Supplementary file 3 [file js9-110-2939-s003.docx]

**Supplementary Table 1** Uni- and multivariable Cox regression models of factors associated with all-cause mortality from 2018 to 2020 in the SEER database

|  | **2018-2020** | | | | | | |  | **2018** | | | | | | |  | **2019** | | | | | | |  | **2020** | | | | | | |
| --- | --- | --- | --- | --- | --- | --- | --- | --- | --- | --- | --- | --- | --- | --- | --- | --- | --- | --- | --- | --- | --- | --- | --- | --- | --- | --- | --- | --- | --- | --- | --- |
|  | **Univariable** | | |  | **Multivariable** | | |  | **Univariable** | | |  | **Multivariable** | | |  | **Univariable** | | |  | **Multivariable** | | |  | **Univariable** | | |  | **Multivariable** | | |
|  | **HR** | **95% CI** | **p-value** |  | **HR** | **95% CI** | **p-value** |  | **HR** | **95% CI** | **p-value** |  | **HR** | **95% CI** | **p-value** |  | **HR** | **95% CI** | **p-value** |  | **HR** | **95% CI** | **p-value** |  | **HR** | **95% CI** | **p-value** |  | **HR** | **95% CI** | **p-value** |
| **Exposure** |  |  |  |  |  |  |  |  |  |  |  |  |  |  |  |  |  |  |  |  |  |  |  |  |  |  |  |  |  |  |  |
| **Year of Diagnosis** |  |  |  |  |  |  |  |  |  |  |  |  |  |  |  |  |  |  |  |  |  |  |  |  |  |  |  |  |  |  |  |
| 2018 | — | — |  |  |  |  |  |  |  |  |  |  |  |  |  |  |  |  |  |  |  |  |  |  |  |  |  |  |  |  |  |
| 2019 | 1.01 | 0.95-1.06 | 0.770 |  |  |  |  |  |  |  |  |  |  |  |  |  |  |  |  |  |  |  |  |  |  |  |  |  |  |  |  |
| 2020 | 1.01 | 0.94-1.08 | 0.823 |  |  |  |  |  |  |  |  |  |  |  |  |  |  |  |  |  |  |  |  |  |  |  |  |  |  |  |  |
| **Demographics** |  |  |  |  |  |  |  |  |  |  |  |  |  |  |  |  |  |  |  |  |  |  |  |  |  |  |  |  |  |  |  |
| **Age** |  |  |  |  |  |  |  |  |  |  |  |  |  |  |  |  |  |  |  |  |  |  |  |  |  |  |  |  |  |  |  |
| < 65y | — | — |  |  | — | — |  |  | — | — |  |  | — | — |  |  | — | — |  |  | — | — |  |  | — | — |  |  | — | — |  |
| ≥ 65y | 2.04 | 1.95-2.15 | < **0.001*** |  | 1.85 | 1.76-1.94 | < **0.001** |  | 1.94 | 1.80-2.08 | < **0.001*** |  | 1.82 | 1.69-1.96 | < **0.001** |  | 2.07 | 1.91-2.24 | < **0.001*** |  | 1.87 | 1.73-2.03 | < **0.001** |  | 2.31 | 2.05-2.61 | < **0.001*** |  | 1.97 | 1.74-2.23 | < **0.001** |
| **Gender** |  |  |  |  |  |  |  |  |  |  |  |  |  |  |  |  |  |  |  |  |  |  |  |  |  |  |  |  |  |  |  |
| Female | — | — |  |  |  |  |  |  | — | — |  |  |  |  |  |  | — | — |  |  |  |  |  |  | — | — |  |  |  |  |  |
| Male | 1.03 | 0.99-1.09 | 0.170 |  |  |  |  |  | 1.03 | 0.95-1.10 | 0.482 |  |  |  |  |  | 1.08 | 0.99-1.17 | 0.068 |  |  |  |  |  | 0.97 | 0.86-1.09 | 0.584 |  |  |  |  |
| **Race** |  |  |  |  |  |  |  |  |  |  |  |  |  |  |  |  |  |  |  |  |  |  |  |  |  |  |  |  |  |  |  |
| Hispanic | — | — |  |  | — | — |  |  | — | — |  |  | — | — |  |  | — | — |  |  |  |  |  |  | — | — |  |  |  |  |  |
| Non-Hispanic | 1.15 | 1.07-1.23 | < **0.001*** |  | 1.21 | 1.13-1.29 | < **0.001** |  | 1.18 | 1.06-1.31 | **0.002*** |  | 1.23 | 1.10-1.37 | < **0.001** |  | 1.11 | 1.00-1.24 | 0.059 |  |  |  |  |  | 1.17 | 0.99-1.39 | 0.068 |  |  |  |  |
| **Median Household Income** |  |  |  |  |  |  |  |  |  |  |  |  |  |  |  |  |  |  |  |  |  |  |  |  |  |  |  |  |  |  |  |
| < $75000 | — | — |  |  | — | — |  |  | — | — |  |  | — | — |  |  | — | — |  |  | — | — |  |  | — | — |  |  | — | — |  |
| ≥ $75000 | 0.87 | 0.83-0.91 | < **0.001*** |  | 1.01 | 0.96-1.07 | 0.621 |  | 0.88 | 0.82-0.95 | < **0.001*** |  | 0.94 | 0.87-1.01 | 0.091 |  | 0.87 | 0.80-0.94 | < **0.001*** |  | 0.99 | 0.91-1.07 | 0.744 |  | 0.82 | 0.73-0.92 | < **0.001*** |  | 1.11 | 0.97-1.28 | 0.143 |
| **Rural/Urban Continuum** |  |  |  |  |  |  |  |  |  |  |  |  |  |  |  |  |  |  |  |  |  |  |  |  |  |  |  |  |  |  |  |
| < 1 million population | — | — |  |  | — | — |  |  | — | — |  |  |  |  |  |  | — | — |  |  |  |  |  |  | — | — |  |  | — | — |  |
| > 1 million population | 0.92 | 0.87-0.97 | **0.002*** |  | 0.92 | 0.87-0.97 | **0.004** |  | 0.95 | 0.88-1.03 | 0.243 |  |  |  |  |  | 0.93 | 0.85-1.01 | 0.100 |  |  |  |  |  | 0.82 | 0.72-0.93 | **0.003*** |  | 0.74 | 0.64-0.85 | < **0.001** |
| **Tumor Features** |  |  |  |  |  |  |  |  |  |  |  |  |  |  |  |  |  |  |  |  |  |  |  |  |  |  |  |  |  |  |  |
| **Tumor Site** |  |  |  |  |  |  |  |  |  |  |  |  |  |  |  |  |  |  |  |  |  |  |  |  |  |  |  |  |  |  |  |
| Supratentorial | — | — |  |  | — | — |  |  | — | — |  |  | — | — |  |  | — | — |  |  | — | — |  |  | — | — |  |  | — | — |  |
| Non-supratentorial | 1.24 | 1.16-1.33 | < **0.001*** |  | 1.08 | 1.00-1.17 | **0.048** |  | 1.21 | 1.09-1.34 | < **0.001*** |  | 1.09 | 0.97-1.22 | 0.140 |  | 1.17 | 1.04-1.31 | **0.008*** |  | 1.01 | 0.89-1.14 | 0.902 |  | 1.51 | 1.30-1.76 | < **0.001*** |  | 1.30 | 1.08-1.56 | **0.006** |
| **Laterality** |  |  |  |  |  |  |  |  |  |  |  |  |  |  |  |  |  |  |  |  |  |  |  |  |  |  |  |  |  |  |  |
| Non-bilateral | — | — |  |  | — | — |  |  | — | — |  |  | — | — |  |  | — | — |  |  | — | — |  |  | — | — |  |  | — | — |  |
| Bilateral | 1.98 | 1.69-2.32 | < **0.001*** |  | 1.81 | 1.54-2.13 | < **0.001** |  | 1.62 | 1.27-2.06 | < **0.001*** |  | 1.55 | 1.21-1.98 | **< 0.001** |  | 2.12 | 1.58-2.85 | < **0.001*** |  | 1.79 | 1.33-2.41 | < **0.001** |  | 2.80 | 2.05-3.81 | < **0.001*** |  | 2.26 | 1.64-3.10 | < **0.001** |
| **Treatment Delay** |  |  |  |  |  |  |  |  |  |  |  |  |  |  |  |  |  |  |  |  |  |  |  |  |  |  |  |  |  |  |  |
| 0 m | — | — |  |  |  |  |  |  | — | — |  |  |  |  |  |  | — | — |  |  |  |  |  |  | — | — |  |  |  |  |  |
| > 0 m | 1.08 | 1.02-1.14 | **0.011** |  |  |  |  |  | 1.09 | 1.00-1.18 | **0.047** |  |  |  |  |  | 1.11 | 1.01-1.21 | **0.033** |  |  |  |  |  | 0.98 | 0.85-1.14 | 0.817 |  |  |  |  |
| **No. of in situ/malignant tumors** |  |  |  |  |  |  |  |  |  |  |  |  |  |  |  |  |  |  |  |  |  |  |  |  |  |  |  |  |  |  |  |
| 1 | — | — |  |  | — | — |  |  | — | — |  |  | — | — |  |  | — | — |  |  | — | — |  |  | — | — |  |  |  |  |  |
| >1 | 1.22 | 1.15-1.29 | < **0.001*** |  | 0.89 | 0.74-1.06 | 0.195 |  | 1.19 | 1.09-1.30 | < **0.001*** |  | 0.97 | 0.75-1.25 | 0.799 |  | 1.32 | 1.20-1.46 | < **0.001*** |  | 0.97 | 0.73-1.29 | 0.835 |  | 1.10 | 0.86-1.27 | 0.185 |  |  |  |  |
| **Primary Lesion** |  |  |  |  |  |  |  |  |  |  |  |  |  |  |  |  |  |  |  |  |  |  |  |  |  |  |  |  |  |  |  |
| Yes | — | — |  |  | — | — |  |  | — | — |  |  | — | — |  |  | — | — |  |  |  |  |  |  | — | — |  |  |  |  |  |
| No | 1.27 | 1.19-1.35 | < **0.001*** |  | 1.22 | 1.01-1.46 | **0.039** |  | 1.25 | 1.14-1.38 | < **0.001*** |  | 1.10 | 0.84-1.43 | 0.502 |  | 1.34 | 1.21-1.48 | < **0.001*** |  | 1.17 | 0.87-1.57 | 0.290 |  | 1.18 | 1.02-1.37 | **0.022** |  |  |  |  |
| **Histological Type** |  |  |  |  |  |  |  |  |  |  |  |  |  |  |  |  |  |  |  |  |  |  |  |  |  |  |  |  |  |  |  |
| GBM subtype | — | — |  |  |  |  |  |  | — | — |  |  |  |  |  |  | — | — |  |  |  |  |  |  | — | — |  |  |  |  |  |
| Non-GBM subtype | 0.97 | 0.84-1.12 | 0.702 |  |  |  |  |  | 1.05 | 0.86-1.27 | 0.633 |  |  |  |  |  | 0.87 | 0.68-1.12 | 0.281 |  |  |  |  |  | 0.97 | 0.69-1.37 | 0.87 |  |  |  |  |
| **Treatment** |  |  |  |  |  |  |  |  |  |  |  |  |  |  |  |  |  |  |  |  |  |  |  |  |  |  |  |  |  |  |  |
| **Surgical Treatment** |  |  |  |  |  |  |  |  |  |  |  |  |  |  |  |  |  |  |  |  |  |  |  |  |  |  |  |  |  |  |  |
| Surgery | — | — |  |  | — | — |  |  | — | — |  |  | — | — |  |  | — | — |  |  | — | — |  |  | — | — |  |  | — | — |  |
| No surgery | 1.60 | 1.48-1.71 | < **0.001*** |  | 1.61 | 1.49-1.73 | < **0.001** |  | 1.68 | 1.51-1.86 | < **0.001*** |  | 1.66 | 1.49-1.85 | < **0.001** |  | 1.53 | 1.36-1.73 | < **0.001*** |  | 1.51 | 1.33-1.72 | < **0.001** |  | 1.54 | 1.29-1.83 | < **0.001*** |  | 1.79 | 1.50-2.15 | < **0.001** |
| **Radiotherapy** |  |  |  |  |  |  |  |  |  |  |  |  |  |  |  |  |  |  |  |  |  |  |  |  |  |  |  |  |  |  |  |
| No | — | — |  |  | — | — |  |  | — | — |  |  | — | — |  |  | — | — |  |  | — | — |  |  | — | — |  |  | — | — |  |
| Yes | 0.55 | 0.52-0.57 | < **0.001*** |  | 0.81 | 0.76-0.85 | < **0.001** |  | 0.61 | 0.57-0.66 | < **0.001*** |  | 0.87 | 0.80-0.95 | **0.002** |  | 0.59 | 0.54-0.64 | < **0.001*** |  | 0.87 | 0.79-0.95 | **0.003** |  | 0.35 | 0.31-0.40 | < **0.001*** |  | 0.58 | 0.50-0.67 | < **0.001** |
| **Chemotherapy** |  |  |  |  |  |  |  |  |  |  |  |  |  |  |  |  |  |  |  |  |  |  |  |  |  |  |  |  |  |  |  |
| No | — | — |  |  | — | — |  |  | — | — |  |  | — | — |  |  | — | — |  |  | — | — |  |  | — | — |  |  | — | — |  |
| Yes | 0.31 | 0.30-0.33 | < **0.001*** |  | 0.37 | 0.35-0.39 | < **0.001** |  | 0.37 | 0.34-0.40 | < **0.001*** |  | 0.41 | 0.37-0.45 | < **0.001** |  | 0.33 | 0.31-0.36 | < **0.001*** |  | 0.39 | 0.36-0.43 | < **0.001** |  | 0.19 | 0.17-0.22 | < **0.001*** |  | 0.27 | 0.24-0.31 | < **0.001** |

*Covariables with a p-value < 0.01 in the univariate Cox regression analysis were added to the multivariable Cox models.

Boldface type indicates statistical significance with two-sided p < 0.05.

Abbreviation: CI, confidence interval; GBM, glioblastoma; m, month (s); HR, hazard ratio; SEER, Surveillance, Epidemiology, and End-Results; y, year (s)
