# Supplementary material for: Glioblastoma patients’ survival and its relevant risk factors during the pre-COVID-19 and post-COVID-19 pandemic: real-world cohort study in the USA and China
Source: Int J Surg. 2024 Feb 19;110(5):2939–49. doi: 10.1097/JS9.0000000000001224 (PMC11093471; doi:10.1097/JS9.0000000000001224)
Supplement: Supplementary file 4 [file js9-110-2939-s004.docx]

**Supplementary Table 2** Uni- and multivariable Cox regression models of factors associated with tumor-specific mortality from 2018 to 2020 in the SEER database

|  | **2018-2020** | | | | | | |  | **2018** | | | | | | |  | **2019** | | | | | | |  | **2020** | | | | | | |
| --- | --- | --- | --- | --- | --- | --- | --- | --- | --- | --- | --- | --- | --- | --- | --- | --- | --- | --- | --- | --- | --- | --- | --- | --- | --- | --- | --- | --- | --- | --- | --- |
|  | **Univariable** | | |  | **Multivariable** | | |  | **Univariable** | | |  | **Multivariable** | | |  | **Univariable** | | |  | **Multivariable** | | |  | **Univariable** | | |  | **Multivariable** | | |
|  | **HR** | **95% CI** | **p-value** |  | **HR** | **95% CI** | **p-value** |  | **HR** | **95% CI** | **p-value** |  | **HR** | **95% CI** | **p-value** |  | **HR** | **95% CI** | **p-value** |  | **HR** | **95% CI** | **p-value** |  | **HR** | **95% CI** | **p-value** |  | **HR** | **95% CI** | **p-value** |
| **Exposure** |  |  |  |  |  |  |  |  |  |  |  |  |  |  |  |  |  |  |  |  |  |  |  |  |  |  |  |  |  |  |  |
| **Year of Diagnosis** |  |  |  |  |  |  |  |  |  |  |  |  |  |  |  |  |  |  |  |  |  |  |  |  |  |  |  |  |  |  |  |
| 2018 | — | — |  |  |  |  |  |  |  |  |  |  |  |  |  |  |  |  |  |  |  |  |  |  |  |  |  |  |  |  |  |
| 2019 | 1.01 | 0.95-1.07 | 0.812 |  |  |  |  |  |  |  |  |  |  |  |  |  |  |  |  |  |  |  |  |  |  |  |  |  |  |  |  |
| 2020 | 0.97 | 0.90-1.05 | 0.479 |  |  |  |  |  |  |  |  |  |  |  |  |  |  |  |  |  |  |  |  |  |  |  |  |  |  |  |  |
| **Demographics** |  |  |  |  |  |  |  |  |  |  |  |  |  |  |  |  |  |  |  |  |  |  |  |  |  |  |  |  |  |  |  |
| **Age** |  |  |  |  |  |  |  |  |  |  |  |  |  |  |  |  |  |  |  |  |  |  |  |  |  |  |  |  |  |  |  |
| < 65y | — | — |  |  | — | — |  |  | — | — |  |  | — | — |  |  | — | — |  |  | — | — |  |  | — | — |  |  | — | — |  |
| ≥ 65y | 2.02 | 1.92-2.12 | < **0.001*** |  | 1.86 | 1.77-1.96 | < **0.001** |  | 1.92 | 1.78-2.06 | < **0.001*** |  | 1.84 | 1.71-1.98 | < **0.001** |  | 2.04 | 1.87-2.21 | < **0.001*** |  | 1.87 | 1.73-2.03 | < **0.001** |  | 2.28 | 2.01-2.60 | < **0.001*** |  | 1.99 | 1.76-2.25 | < **0.001** |
| **Gender** |  |  |  |  |  |  |  |  |  |  |  |  |  |  |  |  |  |  |  |  |  |  |  |  |  |  |  |  |  |  |  |
| Female | — | — |  |  |  |  |  |  | — | — |  |  |  |  |  |  | — | — |  |  |  |  |  |  | — | — |  |  |  |  |  |
| Male | 1.03 | 0.98-1.09 | 0.237 |  |  |  |  |  | 1.03 | 0.96-1.11 | 0.422 |  |  |  |  |  | 1.07 | 0.98-1.16 | 0.135 |  |  |  |  |  | 0.96 | 0.84-1.08 | 0.481 |  |  |  |  |
| **Race** |  |  |  |  |  |  |  |  |  |  |  |  |  |  |  |  |  |  |  |  |  |  |  |  |  |  |  |  |  |  |  |
| Hispanic | — | — |  |  | — | — |  |  | — | — |  |  | — | — |  |  | — | — |  |  |  |  |  |  | — | — |  |  |  |  |  |
| Non-Hispanic | 1.20 | 1.11-1.29 | < **0.001*** |  | 1.22 | 1.14-1.31 | < **0.001** |  | 1.24 | 1.11-1.39 | < **0.001*** |  | 1.25 | 1.12-1.39 | < **0.001** |  | 1.14 | 1.02-1.28 | **0.020** |  |  |  |  |  | 1.22 | 1.02-1.47 | **0.030** |  |  |  |  |
| **Median Household Income** |  |  |  |  |  |  |  |  |  |  |  |  |  |  |  |  |  |  |  |  |  |  |  |  |  |  |  |  |  |  |  |
| < $75000 = 0 | — | — |  |  | — | — |  |  | — | — |  |  | — | — |  |  | — | — |  |  | — | — |  |  | — | — |  |  |  |  |  |
| ≥ $75000 = 1 | 0.88 | 0.83-0.92 | < **0.001*** |  | 1.02 | 0.96-1.07 | 0.606 |  | 0.89 | 0.83-0.96 | **0.003*** |  | 0.94 | 0.87-1.01 | 0.101 |  | 0.86 | 0.79-0.93 | < **0.001*** |  | 0.98 | 0.91-1.07 | 0.692 |  | 0.86 | 0.76-0.97 | **0.017** |  |  |  |  |
| **Rural/Urban Continuum** |  |  |  |  |  |  |  |  |  |  |  |  |  |  |  |  |  |  |  |  |  |  |  |  |  |  |  |  |  |  |  |
| < 1 million population | — | — |  |  | — | — |  |  | — | — |  |  |  |  |  |  | — | — |  |  |  |  |  |  | — | — |  |  | — | — |  |
| > 1 million population | 0.91 | 0.86-0.97 | **0.002*** |  | 0.92 | 0.87-0.98 | **0.005** |  | 0.95 | 0.87-1.04 | 0.249 |  |  |  |  |  | 0.91 | 0.83-1.00 | 0.050 |  |  |  |  |  | 0.83 | 0.72-0.95 | **0.008*** |  | 0.77 | 0.68-0.88 | < **0.001** |
| **Tumor Features** |  |  |  |  |  |  |  |  |  |  |  |  |  |  |  |  |  |  |  |  |  |  |  |  |  |  |  |  |  |  |  |
| **Tumor Site** |  |  |  |  |  |  |  |  |  |  |  |  |  |  |  |  |  |  |  |  |  |  |  |  |  |  |  |  |  |  |  |
| Supratentorial | — | — |  |  | — | — |  |  | — | — |  |  | — | — |  |  | — | — |  |  | — | — |  |  | — | — |  |  | — | — |  |
| Non-supratentorial | 1.25 | 1.16-1.34 | < **0.001*** |  | 1.08 | 1.00-1.17 | 0.058 |  | 1.21 | 1.09-1.35 | < **0.001*** |  | 1.08 | 0.96-1.22 | 0.182 |  | 1.17 | 1.04-1.32 | **0.008*** |  | 1.01 | 0.89-1.14 | 0.902 |  | 1.51 | 1.28-1.77 | < **0.001*** |  | 1.29 | 1.07-1.56 | **0.008** |
| **Laterality** |  |  |  |  |  |  |  |  |  |  |  |  |  |  |  |  |  |  |  |  |  |  |  |  |  |  |  |  |  |  |  |
| Non-bilateral | — | — |  |  | — | — |  |  | — | — |  |  | — | — |  |  | — | — |  |  | — | — |  |  | — | — |  |  | — | — |  |
| Bilateral | 2.10 | 1.78-2.47 | < **0.001*** |  | 1.82 | 1.55-2.14 | < **0.001** |  | 1.70 | 1.33-2.18 | < **0.001*** |  | 1.54 | 1.21-1.98 | **0.001** |  | 2.29 | 1.70-3.08 | < **0.001*** |  | 1.79 | 1.33-2.41 | < **0.001** |  | 2.94 | 2.14-4.06 | < **0.001*** |  | 2.31 | 1.68-3.17 | < **0.001** |
| **Treatment Delay** |  |  |  |  |  |  |  |  |  |  |  |  |  |  |  |  |  |  |  |  |  |  |  |  |  |  |  |  |  |  |  |
| 0 m | — | — |  |  |  |  |  |  | — | — |  |  |  |  |  |  | — | — |  |  |  |  |  |  | — | — |  |  |  |  |  |
| > 0 m | 1.08 | 1.02-1.15 | **0.010** |  |  |  |  |  | 1.09 | 1.00-1.19 | 0.050 |  |  |  |  |  | 1.10 | 1.00-1.21 | 0.054 |  |  |  |  |  | 1.03 | 0.88-1.20 | 0.726 |  |  |  |  |
| **No. of in situ/malignant tumors** |  |  |  |  |  |  |  |  |  |  |  |  |  |  |  |  |  |  |  |  |  |  |  |  |  |  |  |  |  |  |  |
| 1 | — | — |  |  | — | — |  |  | — | — |  |  | — | — |  |  | — | — |  |  | — | — |  |  | — | — |  |  |  |  |  |
| >1 | 1.16 | 1.09-1.24 | < **0.001*** |  | 0.89 | 0.74-1.06 | 0.200 |  | 1.13 | 1.03-1.25 | **0.009*** |  | 0.96 | 0.75-1.25 | 0.779 |  | 1.28 | 1.16-1.42 | < **0.001*** |  | 0.98 | 0.74-1.30 | 0.874 |  | 1.01 | 0.86-1.18 | 0.923 |  |  |  |  |
| **Primary Lesion** |  |  |  |  |  |  |  |  |  |  |  |  |  |  |  |  |  |  |  |  |  |  |  |  |  |  |  |  |  |  |  |
| Yes | — | — |  |  | — | — |  |  | — | — |  |  | — | — |  |  | — | — |  |  |  |  |  |  | — | — |  |  |  |  |  |
| No | 1.21 | 1.13-1.29 | < **0.001** |  | 1.22 | 1.01-1.46 | **0.039** |  | 1.19 | 1.08-1.32 | < **0.001*** |  | 1.10 | 0.84-1.43 | 0.502 |  | 1.30 | 1.17-1.45 | < **0.001*** |  | 1.17 | 0.88-1.57 | 0.287 |  | 1.08 | 0.93-1.27 | 0.320 |  |  |  |  |
| **Histological Type** |  |  |  |  |  |  |  |  |  |  |  |  |  |  |  |  |  |  |  |  |  |  |  |  |  |  |  |  |  |  |  |
| GBM subtype = 0 | — | — |  |  |  |  |  |  | — | — |  |  |  |  |  |  | — | — |  |  |  |  |  |  | — | — |  |  |  |  |  |
| Non-GBM subtype | 0.97 | 0.84-1.12 | 0.670 |  |  |  |  |  | 1.07 | 0.88-1.31 | 0.507 |  |  |  |  |  | 0.87 | 0.67-1.14 | 0.315 |  |  |  |  |  | 0.87 | 0.60-1.28 | 0.489 |  |  |  |  |
| **Treatment** |  |  |  |  |  |  |  |  |  |  |  |  |  |  |  |  |  |  |  |  |  |  |  |  |  |  |  |  |  |  |  |
| **Surgical Treatment** |  |  |  |  |  |  |  |  |  |  |  |  |  |  |  |  |  |  |  |  |  |  |  |  |  |  |  |  |  |  |  |
| Surgery | — | — |  |  | — | — |  |  | — | — |  |  | — | — |  |  | — | — |  |  | — | — |  |  | — | — |  |  | — | — |  |
| No surgery | 1.62 | 1.50-1.75 | < **0.001*** |  | 1.61 | 1.49-1.73 | < **0.001** |  | 1.72 | 1.54-1.92 | < **0.001*** |  | 1.65 | 1.48-1.84 | < **0.001** |  | 1.55 | 1.37-1.76 | < **0.001*** |  | 1.51 | 1.33-1.71 | < **0.001** |  | 1.54 | 1.28-1.85 | < **0.001*** |  | 1.79 | 1.49-2.15 | < **0.001** |
| **Radiotherapy** |  |  |  |  |  |  |  |  |  |  |  |  |  |  |  |  |  |  |  |  |  |  |  |  |  |  |  |  |  |  |  |
| No | — | — |  |  | — | — |  |  | — | — |  |  | — | — |  |  | — | — |  |  | — | — |  |  | — | — |  |  | — | — |  |
| Yes | 0.56 | 0.53-0.59 | < **0.001*** |  | 0.81 | 0.76-0.86 | < **0.001** |  | 0.63 | 0.58-0.68 | < **0.001*** |  | 0.87 | 0.80-0.95 | 0.002 |  | 0.60 | 0.55-0.65 | < **0.001*** |  | 0.87 | 0.79-0.96 | **0.005** |  | 0.36 | 0.32-0.41 | < **0.001*** |  | 0.60 | 0.52-0.69 | < **0.001** |
| **Chemotherapy** |  |  |  |  |  |  |  |  |  |  |  |  |  |  |  |  |  |  |  |  |  |  |  |  |  |  |  |  |  |  |  |
| No | — | — |  |  | — | — |  |  | — | — |  |  | — | — |  |  | — | — |  |  | — | — |  |  | — | — |  |  | — | — |  |
| Yes | 0.32 | 0.30-0.33 | < **0.001*** |  | 0.37 | 0.35-0.39 | < **0.001** |  | 0.38 | 0.35-0.41 | < **0.001*** |  | 0.40 | 0.37-0.44 | < **0.001** |  | 0.33 | 0.31-0.36 | < **0.001*** |  | 0.39 | 0.35-0.43 | < **0.001** |  | 0.19 | 0.17-0.22 | < **0.001*** |  | 0.27 | 0.23-0.31 | < **0.001** |

*Covariables with a p-value < 0.01 in the univariate Cox regression analysis were added to the multivariable Cox models.

Boldface type indicates statistical significance with two-sided p < 0.05.

Abbreviation: CI, confidence interval; GBM, glioblastoma; m, month (s); HR, hazard ratio; SEER, Surveillance, Epidemiology, and End-Results; y, year (s)
