# Supplementary material for: Glioblastoma patients’ survival and its relevant risk factors during the pre-COVID-19 and post-COVID-19 pandemic: real-world cohort study in the USA and China
Source: Int J Surg. 2024 Feb 19;110(5):2939–49. doi: 10.1097/JS9.0000000000001224 (PMC11093471; doi:10.1097/JS9.0000000000001224)
Supplement: Supplementary file 5 [file js9-110-2939-s005.docx]

**Supplementary Table 3** Uni- and multivariable competing risk models of factors associated with mortality from 2018 to 2020 in the SEER database

|  | **2018-2020** | | | | | | |  | **2018** | | | | | | |  | **2019** | | | | | | |  | **2020** | | | | | | |
| --- | --- | --- | --- | --- | --- | --- | --- | --- | --- | --- | --- | --- | --- | --- | --- | --- | --- | --- | --- | --- | --- | --- | --- | --- | --- | --- | --- | --- | --- | --- | --- |
|  | **Unadjusted** | | |  | **Adjusted** | | |  | **Unadjusted** | | |  | **Adjusted** | | |  | **Unadjusted** | | |  | **Adjusted** | | |  | **Unadjusted** | | |  | **Adjusted** | | |
|  | **HR** | **95% CI** | **p-value** |  | **HR** | **95% CI** | **p-value** |  | **HR** | **95% CI** | **p-value** |  | **HR** | **95% CI** | **p-value** |  | **HR** | **95% CI** | **p-value** |  | **HR** | **95% CI** | **p-value** |  | **HR** | **95% CI** | **p-value** |  | **HR** | **95% CI** | **p-value** |
| **Demographics** |  |  |  |  |  |  |  |  |  |  |  |  |  |  |  |  |  |  |  |  |  |  |  |  |  |  |  |  |  |  |  |
| **Age** |  |  |  |  |  |  |  |  |  |  |  |  |  |  |  |  |  |  |  |  |  |  |  |  |  |  |  |  |  |  |  |
| < 65y | — | — |  |  | — | — |  |  | — | — |  |  | — | — |  |  | — | — |  |  | — | — |  |  | — | — |  |  | — | — |  |
| ≥ 65y | 1.93 | 1.83-2.05 | < **0.001*** |  | 1.79 | 1.68-1.90 | < **0.001** |  | 1.76 | 1.61-1.91 | < **0.001*** |  | 1.65 | 1.51-1.81 | < **0.001** |  | 2.03 | 1.84-2.23 | < **0.001*** |  | 1.87 | 1.69-2.07 | < **0.001** |  | 2.38 | 2.05-2.78 | < **0.001*** |  | 2.10 | 1.79-2.46 | < **0.001** |
| **Gender** |  |  |  |  |  |  |  |  |  |  |  |  |  |  |  |  |  |  |  |  |  |  |  |  |  |  |  |  |  |  |  |
| Female | — | — |  |  |  |  |  |  | — | — |  |  |  |  |  |  | — | — |  |  |  |  |  |  | — | — |  |  |  |  |  |
| Male | 1.04 | 0.98-1.11 | 0.160 |  |  |  |  |  | 1.03 | 0.95-1.12 | 0.510 |  |  |  |  |  | 1.09 | 0.99-1.21 | 0.070 |  |  |  |  |  | 0.97 | 0.84-1.12 | 0.680 |  |  |  |  |
| **Race** |  |  |  |  |  |  |  |  |  |  |  |  |  |  |  |  |  |  |  |  |  |  |  |  |  |  |  |  |  |  |  |
| Hispanic | — | — |  |  | — | — |  |  | — | — |  |  | — | — |  |  | — | — |  |  |  |  |  |  | — | — |  |  |  |  |  |
| Non-Hispanic | 1.19 | 1.10-1.29 | < **0.001*** |  | 1.25 | 1.14-1.37 | < **0.001** |  | 1.25 | 1.10-1.41 | < **0.001*** |  | 1.29 | 1.13-1.48 | < **0.001** |  | 1.18 | 1.04-1.34 | **0.012** |  |  |  |  |  | 1.12 | 0.91-1.38 | 0.300 |  |  |  |  |
| **Median Household Income** |  |  |  |  |  |  |  |  |  |  |  |  |  |  |  |  |  |  |  |  |  |  |  |  |  |  |  |  |  |  |  |
| < $75000 | — | — |  |  | — | — |  |  | — | — |  |  |  |  |  |  | — | — |  |  |  |  |  |  | — | — |  |  |  |  |  |
| ≥ $75000 | 0.91 | 0.86-0.97 | **0.002*** |  | 0.97 | 0.91-1.04 | 0.390 |  | 0.93 | 0.86-1.01 | 0.098 |  |  |  |  |  | 0.88 | 0.80-0.97 | **0.010** |  |  |  |  |  | 0.93 | 0.80-1.08 | 0.350 |  |  |  |  |
| **Rural/Urban Continuum** |  |  |  |  |  |  |  |  |  |  |  |  |  |  |  |  |  |  |  |  |  |  |  |  |  |  |  |  |  |  |  |
| < 1 million population | — | — |  |  |  |  |  |  | — | — |  |  |  |  |  |  | — | — |  |  |  |  |  |  | — | — |  |  | — | — |  |
| > 1 million population | 0.93 | 0.87-0.99 | **0.020** |  |  |  |  |  | 0.99 | 0.90-1.08 | 0.740 |  |  |  |  |  | 0.91 | 0.83-1.01 | 0.074 |  |  |  |  |  | 0.82 | 0.70-0.95 | **0.009*** |  | 0.79 | 0.67-0.92 | **0.003** |
| Unknown = 2 |  |  |  |  | - | - | - |  |  |  |  |  |  |  |  |  |  |  |  |  |  |  |  |  |  |  |  |  | - | - | - |
| **Tumor Features** |  |  |  |  |  |  |  |  |  |  |  |  |  |  |  |  |  |  |  |  |  |  |  |  |  |  |  |  |  |  |  |
| **Tumor Site** |  |  |  |  |  |  |  |  |  |  |  |  |  |  |  |  |  |  |  |  |  |  |  |  |  |  |  |  |  |  |  |
| Supratentorial | — | — |  |  | — | — |  |  | — | — |  |  |  |  |  |  | — | — |  |  |  |  |  |  | — | — |  |  | — | — |  |
| Non-supratentorial | 1.16 | 1.06-1.29 | **0.002*** |  | 1.04 | 0.94-1.16 | 0.470 |  | 1.08 | 0.94-1.25 | 0.280 |  |  |  |  |  | 1.14 | 0.98-1.32 | 0.097 |  |  |  |  |  | 1.50 | 1.21-1.86 | < **0.001*** |  | 1.27 | 1.00-1.60 | **0.046** |
| Unknown |  |  |  |  |  |  |  |  |  |  |  |  |  |  |  |  |  |  |  |  |  |  |  |  |  |  |  |  |  |  |  |
| **Laterality** |  |  |  |  |  |  |  |  |  |  |  |  |  |  |  |  |  |  |  |  |  |  |  |  |  |  |  |  |  |  |  |
| Non-bilateral | — | — |  |  | — | — |  |  | — | — |  |  | — | — |  |  | — | — |  |  | — | — |  |  | — | — |  |  |  |  |  |
| Bilateral | 1.94 | 1.53-2.46 | < **0.001*** |  | 1.75 | 1.39-2.21 | < **0.001** |  | 1.65 | 1.13-2.40 | < **0.001*** |  | 1.43 | 0.97-2.10 | 0.070 |  | 2.18 | 1.50-3.18 | < **0.001*** |  | 1.77 | 1.19-2.62 | **0.005** |  | 2.36 | 1.54-3.61 | < **0.001*** |  | 2.20 | 1.31-3.67 | **0.003** |
| Unknown |  |  |  |  |  |  |  |  |  |  |  |  |  |  |  |  |  |  |  |  |  |  |  |  |  |  |  |  |  |  |  |
| **Treatment Delay** |  |  |  |  |  |  |  |  |  |  |  |  |  |  |  |  |  |  |  |  |  |  |  |  |  |  |  |  |  |  |  |
| 0 m | — | — |  |  |  |  |  |  | — | — |  |  |  |  |  |  | — | — |  |  |  |  |  |  | — | — |  |  |  |  |  |
| > 0 m | 1.07 | 1.00-1.15 | **0.040** |  |  |  |  |  | 1.08 | 0.98-1.20 | 0.120 |  |  |  |  |  | 1.07 | 0.96-1.20 | 0.230 |  |  |  |  |  | 1.04 | 0.87-1.26 | 0.690 |  |  |  |  |
| **No. of in situ/malignant tumors** |  |  |  |  |  |  |  |  |  |  |  |  |  |  |  |  |  |  |  |  |  |  |  |  |  |  |  |  |  |  |  |
| 1 | — | — |  |  |  |  |  |  | — | — |  |  |  |  |  |  | — | — |  |  | — | — |  |  | — | — |  |  |  |  |  |
| >1 | 1.09 | 1.01-1.17 | **0.020** |  |  |  |  |  | 1.03 | 0.92-1.15 | 0.600 |  |  |  |  |  | 1.25 | 1.11-1.41 | < **0.001*** |  | 0.75 | 0.47-1.20 | 0.240 |  | 0.97 | 0.81-1.17 | 0.770 |  |  |  |  |
| **Primary Lesion** |  |  |  |  |  |  |  |  |  |  |  |  |  |  |  |  |  |  |  |  |  |  |  |  |  |  |  |  |  |  |  |
| Yes | — | — |  |  | — | — |  |  | — | — |  |  |  |  |  |  | — | — |  |  | — | — |  |  | — | — |  |  |  |  |  |
| No | 1.14 | 1.06-1.23 | < **0.001*** |  | 0.97 | 0.89-1.05 | 0.400 |  | 1.08 | 0.96-1.22 | 0.190 |  |  |  |  |  | 1.29 | 1.14-1.45 | < **0.001*** |  | 1.41 | 0.87-2.28 | 0.160 |  | 1.05 | 0.87-1.27 | 0.620 |  |  |  |  |
| **Histological Type** |  |  |  |  |  |  |  |  |  |  |  |  |  |  |  |  |  |  |  |  |  |  |  |  |  |  |  |  |  |  |  |
| GBM subtype | — | — |  |  |  |  |  |  | — | — |  |  | — | — |  |  | — | — |  |  |  |  |  |  | — | — |  |  | — | — |  |
| Non GBM subtype | 0.96 | 0.81-1.14 | 0.640 |  |  |  |  |  | 1.10 | 0.88-1.37 | 0.400 |  |  |  |  |  | 0.79 | 0.57-1.10 | 0.160 |  |  |  |  |  | 0.95 | 0.62-1.47 | 0.820 |  |  |  |  |
| **Treatment** |  |  |  |  |  |  |  |  |  |  |  |  |  |  |  |  |  |  |  |  |  |  |  |  |  |  |  |  |  |  |  |
| **Surgical Treatment** |  |  |  |  |  |  |  |  |  |  |  |  |  |  |  |  |  |  |  |  |  |  |  |  |  |  |  |  |  |  |  |
| Surgery | — | — |  |  | — | — |  |  | — | — |  |  | — | — |  |  | — | — |  |  | — | — |  |  | — | — |  |  | — | — |  |
| No surgery | 1.59 | 1.45-1.74 | < **0.001*** |  | 1.64 | 1.48-1.82 | < **0.001** |  | 1.72 | 1.51-1.96 | < **0.001*** |  | 1.77 | 1.54-2.04 | < **0.001** |  | 1.52 | 1.30-1.79 | < **0.001*** |  | 1.58 | 1.32-1.88 | < **0.001** |  | 1.41 | 1.11-1.79 | **0.005** |  | 1.63 | 1.27-2.10 | < **0.001** |
| Unknown |  |  |  |  | - | - | - |  |  |  |  |  | - | - | - |  |  |  |  |  | - | - | - |  |  |  |  |  | - | - | - |
| **Radiotherapy** |  |  |  |  |  |  |  |  |  |  |  |  |  |  |  |  |  |  |  |  |  |  |  |  |  |  |  |  |  |  |  |
| No | — | — |  |  | — | — |  |  | — | — |  |  | — | — |  |  | — | — |  |  | — | — |  |  | — | — |  |  | — | — |  |
| Yes | 0.60 | 0.56-0.63 | < **0.001*** |  | 0.84 | 0.78-0.90 | < **0.001** |  | 0.65 | 0.60-0.72 | < **0.001*** |  | 0.84 | 0.75-0.93 | **0.001** |  | 0.65 | 0.59-0.71 | < **0.001*** |  | 0.89 | 0.79-0.97 | **0.042** |  | 0.37 | 0.32-0.43 | < **0.001** |  | 0.65 | 0.55-0.76 | < **0.001** |
| **Chemotherapy** |  |  |  |  |  |  |  |  |  |  |  |  |  |  |  |  |  |  |  |  |  |  |  |  |  |  |  |  |  |  |  |
| No | — | — |  |  | — | — |  |  | — | — |  |  | — | — |  |  | — | — |  |  | — | — |  |  | — | — |  |  | — | — |  |
| Yes | 0.38 | 0.35-0.40 | < **0.001*** |  | 0.42 | 0.39-0.46 | < **0.001** |  | 0.46 | 0.41-0.51 | < **0.001*** |  | 0.50 | 0.44-0.57 | < **0.001** |  | 0.39 | 0.35-0.44 | < **0.001*** |  | 0.44 | 0.39-0.49 | < **0.001** |  | 0.21 | 0.18-0.24 | < **0.001** |  | 0.26 | 0.23-0.31 | < **0.001** |

*Covariables with a p-value < 0.01 in the competing risk analysis were added to the multivariable competing risk models.

Boldface type indicates statistical significance with two-sided p < 0.05.

Abbreviation: CI, confidence interval; GBM, glioblastoma; m, month (s); HR, hazard ratio; SEER, Surveillance, Epidemiology, and End-Results; y, year (s)
