# Supplementary material for: Glioblastoma patients’ survival and its relevant risk factors during the pre-COVID-19 and post-COVID-19 pandemic: real-world cohort study in the USA and China
Source: Int J Surg. 2024 Feb 19;110(5):2939–49. doi: 10.1097/JS9.0000000000001224 (PMC11093471; doi:10.1097/JS9.0000000000001224)
Supplement: Supplementary file 6 [file js9-110-2939-s006.docx]

**Supplementary Table 4** Uni- and multivariable Cox regression models of factors associated with all-cause mortality from 2018 to 2020 in the CGC

|  | **2018-2020** | | | | | | |  | **2018** | | | | | | |  | **2019** | | | | | | |  | **2020** | | | | | | |
| --- | --- | --- | --- | --- | --- | --- | --- | --- | --- | --- | --- | --- | --- | --- | --- | --- | --- | --- | --- | --- | --- | --- | --- | --- | --- | --- | --- | --- | --- | --- | --- |
|  | **Univariable** | | |  | **Multivariable** | | |  | **Univariable** | | |  | **Multivariable** | | |  | **Univariable** | | |  | **Multivariable** | | |  | **Univariable** | | |  | **Multivariable** | | |
|  | **HR** | **95% CI** | **p-value** |  | **HR** | **95% CI** | **p-value** |  | **HR** | **95% CI** | **p-value** |  | **HR** | **95% CI** | **p-value** |  | **HR** | **95% CI** | **p-value** |  | **HR** | **95% CI** | **p-value** |  | **HR** | **95% CI** | **p-value** |  | **HR** | **95% CI** | **p-value** |
| **Exposure** |  |  |  |  |  |  |  |  |  |  |  |  |  |  |  |  |  |  |  |  |  |  |  |  |  |  |  |  |  |  |  |
| **Year of Diagnosis** |  |  |  |  |  |  |  |  |  |  |  |  |  |  |  |  |  |  |  |  |  |  |  |  |  |  |  |  |  |  |  |
| 2018 | — | — |  |  |  |  |  |  |  |  |  |  |  |  |  |  |  |  |  |  |  |  |  |  |  |  |  |  |  |  |  |
| 2019 | 0.90 | 0.64-1.26 | 0.547 |  |  |  |  |  |  |  |  |  |  |  |  |  |  |  |  |  |  |  |  |  |  |  |  |  |  |  |  |
| 2020 | 0.93 | 0.59-1.45 | 0.740 |  |  |  |  |  |  |  |  |  |  |  |  |  |  |  |  |  |  |  |  |  |  |  |  |  |  |  |  |
| **Demographics** |  |  |  |  |  |  |  |  |  |  |  |  |  |  |  |  |  |  |  |  |  |  |  |  |  |  |  |  |  |  |  |
| **Age** |  |  |  |  |  |  |  |  |  |  |  |  |  |  |  |  |  |  |  |  |  |  |  |  |  |  |  |  |  |  |  |
| < 65y | — | — |  |  |  |  |  |  | — | — |  |  |  |  |  |  | — | — |  |  |  |  |  |  | — | — |  |  |  |  |  |
| ≥ 65y | 1.28 | 0.83-1.97 | 0.271 |  |  |  |  |  | 1.45 | 0.65-3.20 | 0.364 |  |  |  |  |  | 1.19 | 0.64-2.20 | 0.581 |  |  |  |  |  | 1.42 | 0.53-3.77 | 0.485 |  |  |  |  |
| **Gender** |  |  |  |  |  |  |  |  |  |  |  |  |  |  |  |  |  |  |  |  |  |  |  |  |  |  |  |  |  |  |  |
| Female | — | — |  |  |  |  |  |  | — | — |  |  |  |  |  |  | — | — |  |  |  |  |  |  | — | — |  |  |  |  |  |
| Male | 1.14 | 0.83-1.57 | 0.419 |  |  |  |  |  | 1.31 | 0.77-2.23 | 0.323 |  |  |  |  |  | 1.18 | 0.74-1.87 | 0.487 |  |  |  |  |  | 0.68 | 0.30-1.51 | 0.342 |  |  |  |  |
| **Race** |  |  |  |  |  |  |  |  |  |  |  |  |  |  |  |  |  |  |  |  |  |  |  |  |  |  |  |  |  |  |  |
| Han | — | — |  |  |  |  |  |  | — | — |  |  |  |  |  |  | — | — |  |  |  |  |  |  | — | — |  |  |  |  |  |
| Non-Han | 2.56 | 0.36-18.41 | 0.351 |  |  |  |  |  | — | — | — |  |  |  |  |  | 2.33 | 0.32-17.02 | 0.403 |  |  |  |  |  | — | — | — |  |  |  |  |
| **Residency** |  |  |  |  |  |  |  |  |  |  |  |  |  |  |  |  |  |  |  |  |  |  |  |  |  |  |  |  |  |  |  |
| Rural | — | — |  |  |  |  |  |  | — | — |  |  |  |  |  |  | — | — |  |  |  |  |  |  | — | — |  |  |  |  |  |
| Urban | 0.76 | 0.56-1.02 | 0.071 |  |  |  |  |  | 0.69 | 0.42-1.15 | 0.152 |  |  |  |  |  | 0.70 | 0.45-1.10 | 0.120 |  |  |  |  |  | 1.02 | 0.47-2.19 | 0.967 |  |  |  |  |
| **Tumor Features** |  |  |  |  |  |  |  |  |  |  |  |  |  |  |  |  |  |  |  |  |  |  |  |  |  |  |  |  |  |  |  |
| **Tumor Site** |  |  |  |  |  |  |  |  |  |  |  |  |  |  |  |  |  |  |  |  |  |  |  |  |  |  |  |  |  |  |  |
| Supratentorial | — | — |  |  |  |  |  |  | — | — |  |  |  |  |  |  | — | — |  |  |  |  |  |  | — | — |  |  | — | — |  |
| Non-supratentorial | 0.51 | 0.16-1.62 | 0.257 |  |  |  |  |  | 5.72 | 0.75-43.68 | 0.093 |  |  |  |  |  | — | — | — |  |  |  |  |  | 41.78 | 2.59-673.13 | **0.008*** |  | 41.78 | 2.59-673.13 | **0.008*** |
| **Laterality** |  |  |  |  |  |  |  |  |  |  |  |  |  |  |  |  |  |  |  |  |  |  |  |  |  |  |  |  |  |  |  |
| Non-bilateral = | — | — |  |  |  |  |  |  | — | — |  |  |  |  |  |  | — | — |  |  |  |  |  |  | — | — |  |  |  |  |  |
| Bilateral | 0.91 | 0.66-1.24 | 0.536 |  |  |  |  |  | 1.05 | 0.61-1.80 | 0.864 |  |  |  |  |  | 0.70 | 0.44-1.10 | 0.122 |  |  |  |  |  | 1.42 | 0.64-3.15 | 0.386 |  |  |  |  |
| **No. of in situ/malignant tumors** |  |  |  |  |  |  |  |  |  |  |  |  |  |  |  |  |  |  |  |  |  |  |  |  |  |  |  |  |  |  |  |
| 1 | — | — |  |  | — | — |  |  | — | — |  |  | — | — |  |  | — | — |  |  | — | — |  |  | — | — |  |  |  |  |  |
| >1 | 2.03 | 1.37-3.03 | < **0.001*** |  | — | — | — |  | 2.16 | 1.21-3.84 | **0.009*** |  | — | — | — |  | 2.61 | 1.34-5.11 | **0.005*** |  | — | — | — |  | 0.79 | 0.24-2.66 | 0.709 |  |  |  |  |
| **Primary Lesion** |  |  |  |  |  |  |  |  |  |  |  |  |  |  |  |  |  |  |  |  |  |  |  |  |  |  |  |  |  |  |  |
| Yes | — | — |  |  | — | — |  |  | — | — |  |  | — | — |  |  | — | — |  |  | — | — |  |  | — | — |  |  |  |  |  |
| No | 2.03 | 1.37-3.03 | < **0.001*** |  | 2.09 | 1.40-3.11 | < **0.001** |  | 2.16 | 1.21-3.84 | **0.009*** |  | 2.16 | 1.21-3.84 | **0.009*** |  | 2.61 | 1.34-5.11 | **0.005*** |  | 2.63 | 1.31-5.27 | **0.006** |  | 0.79 | 0.24-2.66 | 0.709 |  |  |  |  |
| **Histological Type** |  |  |  |  |  |  |  |  |  |  |  |  |  |  |  |  |  |  |  |  |  |  |  |  |  |  |  |  |  |  |  |
| GBM subtype | — | — |  |  |  |  |  |  | — | — |  |  |  |  |  |  | — | — |  |  |  |  |  |  | — | — |  |  |  |  |  |
| Non-GBM subtype | 1.90 | 0.70-5.17 | 0.206 |  |  |  |  |  | 1.22 | 0.29-5.04 | 0.787 |  |  |  |  |  | 3.23 | 0.78-13.36 | 0.105 |  |  |  |  |  | — | — | — |  |  |  |  |
| **Treatment** |  |  |  |  |  |  |  |  |  |  |  |  |  |  |  |  |  |  |  |  |  |  |  |  |  |  |  |  |  |  |  |
| **Surgical Treatment** |  |  |  |  |  |  |  |  |  |  |  |  |  |  |  |  |  |  |  |  |  |  |  |  |  |  |  |  |  |  |  |
| Surgery | — | — |  |  | — | — |  |  | — | — |  |  |  |  |  |  | — | — |  |  |  |  |  |  | — | — |  |  |  |  |  |
| No surgery | 14.85 | 4.51-48.90 | < **0.001*** |  | 17.00 | 5.14-56.17 | < **0.001** |  | 5.36 | 0.70-40.75 | 0.105 |  |  |  |  |  | — | — | — |  |  |  |  |  | — | — | — |  |  |  |  |
| **Radiotherapy** |  |  |  |  |  |  |  |  |  |  |  |  |  |  |  |  |  |  |  |  |  |  |  |  |  |  |  |  |  |  |  |
| No | — | — |  |  |  |  |  |  | — | — |  |  |  |  |  |  | — | — |  |  |  |  |  |  | — | — |  |  | — | — |  |
| Yes | 0.92 | 0.62-1.37 | 0.690 |  |  |  |  |  | 1.01 | 0.53-1.91 | 0.977 |  |  |  |  |  | 0.89 | 0.52-1.54 | 0.681 |  |  |  |  |  | — | — | — |  |  |  |  |
| **Chemotherapy** |  |  |  |  |  |  |  |  |  |  |  |  |  |  |  |  |  |  |  |  |  |  |  |  |  |  |  |  |  |  |  |
| No | — | — |  |  |  |  |  |  | — | — |  |  |  |  |  |  | — | — |  |  |  |  |  |  | — | — |  |  | — | — |  |
| Yes | 0.98 | 0.66-1.47 | 0.932 |  |  |  |  |  | 0.96 | 0.50-1.86 | 0.911 |  |  |  |  |  | 1.01 | 0.59-1.75 | 0.958 |  |  |  |  |  | — | — | — |  |  |  |  |

*Covariables with a p-value < 0.01 in the univariate Cox regression analysis were added to the multivariable Cox models.

Boldface type indicates statistical significance with two-sided p < 0.05.

Abbreviation: CI, confidence interval; GBM, glioblastoma; m, month (s); HR, hazard ratio
