# Supplementary material for: Glioblastoma patients’ survival and its relevant risk factors during the pre-COVID-19 and post-COVID-19 pandemic: real-world cohort study in the USA and China
Source: Int J Surg. 2024 Feb 19;110(5):2939–49. doi: 10.1097/JS9.0000000000001224 (PMC11093471; doi:10.1097/JS9.0000000000001224)
Supplement: Supplementary file 7 [file js9-110-2939-s007.docx]

**Supplementary Table 5** Uni- and multivariable Cox regression models of factors associated with tumor-specific mortality from 2018 to 2020 in the CGC

|  | **2018-2020** | | | | | | |  | **2018** | | | | | | |  | **2019** | | | | | | |  | **2020** | | | | | | |
| --- | --- | --- | --- | --- | --- | --- | --- | --- | --- | --- | --- | --- | --- | --- | --- | --- | --- | --- | --- | --- | --- | --- | --- | --- | --- | --- | --- | --- | --- | --- | --- |
|  | **Univariable** | | |  | **Multivariable** | | |  | **Univariable** | | |  | **Multivariable** | | |  | **Univariable** | | |  | **Multivariable** | | |  | **Univariable** | | |  | **Multivariable** | | |
|  | **HR** | **95% CI** | **p-value** |  | **HR** | **95% CI** | **p-value** |  | **HR** | **95% CI** | **p-value** |  | **HR** | **95% CI** | **p-value** |  | **HR** | **95% CI** | **p-value** |  | **HR** | **95% CI** | **p-value** |  | **HR** | **95% CI** | **p-value** |  | **HR** | **95% CI** | **p-value** |
| **Exposure** |  |  |  |  |  |  |  |  |  |  |  |  |  |  |  |  |  |  |  |  |  |  |  |  |  |  |  |  |  |  |  |
| **Year of Diagnosis** |  |  |  |  |  |  |  |  |  |  |  |  |  |  |  |  |  |  |  |  |  |  |  |  |  |  |  |  |  |  |  |
| 2018 | — | — |  |  |  |  |  |  |  |  |  |  |  |  |  |  |  |  |  |  |  |  |  |  |  |  |  |  |  |  |  |
| 2019 | 0.91 | 0.65-1.27 | 0.577 |  |  |  |  |  |  |  |  |  |  |  |  |  |  |  |  |  |  |  |  |  |  |  |  |  |  |  |  |
| 2020 | 0.94 | 0.60-1.48 | 0.787 |  |  |  |  |  |  |  |  |  |  |  |  |  |  |  |  |  |  |  |  |  |  |  |  |  |  |  |  |
| **Demographics** |  |  |  |  |  |  |  |  |  |  |  |  |  |  |  |  |  |  |  |  |  |  |  |  |  |  |  |  |  |  |  |
| **Age** |  |  |  |  |  |  |  |  |  |  |  |  |  |  |  |  |  |  |  |  |  |  |  |  |  |  |  |  |  |  |  |
| < 65y | — | — |  |  |  |  |  |  | — | — |  |  |  |  |  |  | — | — |  |  |  |  |  |  | — | — |  |  |  |  |  |
| ≥ 65y | 1.23 | 0.79-1.91 | 0.360 |  |  |  |  |  | 1.24 | 0.53-2.89 | 0.626 |  |  |  |  |  | 1.21 | 0.65-2.23 | 0.549 |  |  |  |  |  | 1.42 | 0.53-3.77 | 0.485 |  |  |  |  |
| **Gender** |  |  |  |  |  |  |  |  |  |  |  |  |  |  |  |  |  |  |  |  |  |  |  |  |  |  |  |  |  |  |  |
| Female | — | — |  |  |  |  |  |  | — | — |  |  |  |  |  |  | — | — |  |  |  |  |  |  | — | — |  |  |  |  |  |
| Male | 1.15 | 0.83-1.58 | 0.396 |  |  |  |  |  | 1.36 | 0.80-2.34 | 0.259 |  |  |  |  |  | 1.16 | 0.73-1.84 | 0.536 |  |  |  |  |  | 0.68 | 0.30-1.51 | 0.342 |  |  |  |  |
| **Race** |  |  |  |  |  |  |  |  |  |  |  |  |  |  |  |  |  |  |  |  |  |  |  |  |  |  |  |  |  |  |  |
| Han | — | — |  |  |  |  |  |  | — | — |  |  |  |  |  |  | — | — |  |  |  |  |  |  | — | — |  |  |  |  |  |
| Non-Han | 2.59 | 0.36-18.63 | 0.345 |  |  |  |  |  | — | — | — |  |  |  |  |  | 2.39 | 0.33-17.44 | 0.390 |  |  |  |  |  | — | — | — |  |  |  |  |
| **Residency** |  |  |  |  |  |  |  |  |  |  |  |  |  |  |  |  |  |  |  |  |  |  |  |  |  |  |  |  |  |  |  |
| Rural | — | — |  |  |  |  |  |  | — | — |  |  |  |  |  |  | — | — |  |  |  |  |  |  | — | — |  |  |  |  |  |
| Urban | 0.76 | 0.56-1.02 | 0.070 |  |  |  |  |  | 0.67 | 0.40-1.12 | 0.125 |  |  |  |  |  | 0.72 | 0.46-1.12 | 0.142 |  |  |  |  |  | 1.02 | 0.47-2.19 | 0.967 |  |  |  |  |
| **Tumor Features** |  |  |  |  |  |  |  |  |  |  |  |  |  |  |  |  |  |  |  |  |  |  |  |  |  |  |  |  |  |  |  |
| **Tumor Site** |  |  |  |  |  |  |  |  |  |  |  |  |  |  |  |  |  |  |  |  |  |  |  |  |  |  |  |  |  |  |  |
| Supratentorial | — | — |  |  |  |  |  |  | — | — |  |  |  |  |  |  | — | — |  |  |  |  |  |  | — | — |  |  | — | — |  |
| Non-supratentorial | 0.52 | 0.17-1.65 | 0.269 |  |  |  |  |  | 5.74 | 0.75-43.83 | 0.092 |  |  |  |  |  | — | — | — |  |  |  |  |  | 41.78 | 2.59-673.13 | **0.008*** |  | 41.78 | 2.59-673.13 | **0.008*** |
| **Laterality** |  |  |  |  |  |  |  |  |  |  |  |  |  |  |  |  |  |  |  |  |  |  |  |  |  |  |  |  |  |  |  |
| Non-bilateral | — | — |  |  |  |  |  |  | — | — |  |  |  |  |  |  | — | — |  |  |  |  |  |  | — | — |  |  |  |  |  |
| Bilateral | 0.93 | 0.68-1.28 | 0.662 |  |  |  |  |  | 1.09 | 0.63-1.88 | 0.755 |  |  |  |  |  | 0.72 | 0.45-1.13 | 0.153 |  |  |  |  |  | 1.42 | 0.64-3.15 | 0.386 |  |  |  |  |
| **No. of in situ/malignant tumors** |  |  |  |  |  |  |  |  |  |  |  |  |  |  |  |  |  |  |  |  |  |  |  |  |  |  |  |  |  |  |  |
| 1 | — | — |  |  | — | — |  |  | — | — |  |  | — | — |  |  | — | — |  |  | — | — |  |  | — | — |  |  |  |  |  |
| >1 | 2.06 | 1.39-3.07 | < **0.001*** |  | — | — | — |  | 2.19 | 1.23-3.90 | **0.008*** |  | — | — | — |  | 2.68 | 1.37-5.23 | **0.004*** |  | — | — | — |  | 0.79 | 0.24-2.66 | 0.709 |  |  |  |  |
| **Primary Lesion** |  |  |  |  |  |  |  |  |  |  |  |  |  |  |  |  |  |  |  |  |  |  |  |  |  |  |  |  |  |  |  |
| Yes | — | — |  |  | — | — |  |  | — | — |  |  | — | — |  |  | — | — |  |  | — | — |  |  | — | — |  |  |  |  |  |
| No | 2.06 | 1.39-3.07 | < **0.001*** |  | 2.10 | 1.41-3.12 | < **0.001** |  | 2.19 | 1.23-3.90 | **0.008*** |  | 2.19 | 1.23-3.90 | **0.008*** |  | 2.68 | 1.37-5.23 | **0.004*** |  | 2.68 | 1.37-5.23 | **0.004*** |  | 0.79 | 0.24-2.66 | 0.709 |  |  |  |  |
| **Histological Type** |  |  |  |  |  |  |  |  |  |  |  |  |  |  |  |  |  |  |  |  |  |  |  |  |  |  |  |  |  |  |  |
| GBM subtype | — | — |  |  |  |  |  |  | — | — |  |  |  |  |  |  | — | — |  |  |  |  |  |  | — | — |  |  |  |  |  |
| Non GBM subtype | 1.93 | 0.71-5.23 | 0.198 |  |  |  |  |  | 1.22 | 0.29-5.04 | 0.787 |  |  |  |  |  | 3.34 | 0.81-13.82 | 0.096 |  |  |  |  |  | — | — | — |  |  |  |  |
| **Treatment** |  |  |  |  |  |  |  |  |  |  |  |  |  |  |  |  |  |  |  |  |  |  |  |  |  |  |  |  |  |  |  |
| **Surgical Treatment** |  |  |  |  |  |  |  |  |  |  |  |  |  |  |  |  |  |  |  |  |  |  |  |  |  |  |  |  |  |  |  |
| Surgery | — | — |  |  | — | — |  |  | — | — |  |  |  |  |  |  | — | — |  |  |  |  |  |  | — | — |  |  |  |  |  |
| No surgery | 15.54 | 4.71-51.27 | < **0.001*** |  | 17.02 | 5.15-56.24 | < **0.001** |  | 5.36 | 0.70-40.75 | 0.105 |  |  |  |  |  | — | — | — |  |  |  |  |  | — | — | — |  |  |  |  |
| **Radiotherapy** |  |  |  |  |  |  |  |  |  |  |  |  |  |  |  |  |  |  |  |  |  |  |  |  |  |  |  |  |  |  |  |
| No | — | — |  |  |  |  |  |  | — | — |  |  |  |  |  |  | — | — |  |  |  |  |  |  | — | — |  |  | — | — |  |
| Yes | 0.90 | 0.60-1.35 | 0.603 |  |  |  |  |  | 1.03 | 0.54-1.95 | 0.933 |  |  |  |  |  | 0.83 | 0.48-1.46 | 0.528 |  |  |  |  |  | — | — | — |  |  |  |  |
| **Chemotherapy** |  |  |  |  |  |  |  |  |  |  |  |  |  |  |  |  |  |  |  |  |  |  |  |  |  |  |  |  |  |  |  |
| No | — | — |  |  |  |  |  |  | — | — |  |  |  |  |  |  | — | — |  |  |  |  |  |  | — | — |  |  | — | — |  |
| Yes | 0.96 | 0.63-1.44 | 0.831 |  |  |  |  |  | 0.99 | 0.51-1.91 | 0.977 |  |  |  |  |  | 0.95 | 0.54-1.67 | 0.861 |  |  |  |  |  | — | — | — |  |  |  |  |

*Covariables with a p-value < 0.01 in the univariate Cox regression analysis were added to the multivariable Cox models.

Boldface type indicates statistical significance with two-sided p < 0.05.

Abbreviation: CI, confidence interval; GBM, glioblastoma; m, month (s); HR, hazard ratio
