# Supplementary material for: Glioblastoma patients’ survival and its relevant risk factors during the pre-COVID-19 and post-COVID-19 pandemic: real-world cohort study in the USA and China
Source: Int J Surg. 2024 Feb 19;110(5):2939–49. doi: 10.1097/JS9.0000000000001224 (PMC11093471; doi:10.1097/JS9.0000000000001224)
Supplement: Supplementary file 8 [file js9-110-2939-s008.docx]

**Supplementary Table 6** Uni- and multivariable competing risk models of factors associated with mortality from 2018 to 2020 in the CGC database

|  | **2018-2020** | | | | | | |  | **2018** | | | | | | |  | **2019** | | | | | | |  | **2020** | | | | | | |
| --- | --- | --- | --- | --- | --- | --- | --- | --- | --- | --- | --- | --- | --- | --- | --- | --- | --- | --- | --- | --- | --- | --- | --- | --- | --- | --- | --- | --- | --- | --- | --- |
|  | **Unadjusted** | | |  | **Adjusted** | | |  | **Unadjusted** | | |  | **Adjusted** | | |  | **Unadjusted** | | |  | **Adjusted** | | |  | **Unadjusted** | | |  | **Adjusted** | | |
|  | **HR** | **95% CI** | **p-value** |  | **HR** | **95% CI** | **p-value** |  | **HR** | **95% CI** | **p-value** |  | **HR** | **95% CI** | **p-value** |  | **HR** | **95% CI** | **p-value** |  | **HR** | **95% CI** | **p-value** |  | **HR** | **95% CI** | **p-value** |  | **HR** | **95% CI** | **p-value** |
| **Demographics** |  |  |  |  |  |  |  |  |  |  |  |  |  |  |  |  |  |  |  |  |  |  |  |  |  |  |  |  |  |  |  |
| **Age** |  |  |  |  |  |  |  |  |  |  |  |  |  |  |  |  |  |  |  |  |  |  |  |  |  |  |  |  |  |  |  |
| < 65y | — | — |  |  |  |  |  |  | — | — |  |  |  |  |  |  | — | — |  |  |  |  |  |  | — | — |  |  | — | — |  |
| ≥ 65y | 1.16 | 0.79-1.72 | 0.450 |  |  |  |  |  | 1.08 | 0.43-2.69 | 0.880 |  |  |  |  |  | 1.18 | 0.73-1.89 | 0.500 |  |  |  |  |  | 1.43 | 0.59-3.48 | 0.430 |  |  |  |  |
| **Gender** |  |  |  |  |  |  |  |  |  |  |  |  |  |  |  |  |  |  |  |  |  |  |  |  |  |  |  |  |  |  |  |
| Female | — | — |  |  |  |  |  |  | — | — |  |  |  |  |  |  | — | — |  |  |  |  |  |  | — | — |  |  |  |  |  |
| Male | 1.17 | 0.85-1.59 | 0.340 |  |  |  |  |  | 1.39 | 0.82-2.36 | 0.220 |  |  |  |  |  | 1.17 | 0.76-1.81 | 0.480 |  |  |  |  |  | 0.69 | 0.32-1.47 | 0.330 |  |  |  |  |
| **Race** |  |  |  |  |  |  |  |  |  |  |  |  |  |  |  |  |  |  |  |  |  |  |  |  |  |  |  |  |  |  |  |
| Han | — | — |  |  | — | — |  |  | — | — |  |  |  |  |  |  | — | — |  |  | — | — |  |  | — | — |  |  |  |  |  |
| Non-Han | 2.44 | 1.95-3.04 | < **0.001*** |  | 2.85 | 2.23-3.65 | < **0.001** |  | — | — | — |  |  |  |  |  | 2.15 | 1.57-2.94 | < **0.001*** |  | 2.41 | 1.72-3.37 | < **0.001** |  | — | — | — |  |  |  |  |
| **Residency** |  |  |  |  |  |  |  |  |  |  |  |  |  |  |  |  |  |  |  |  |  |  |  |  |  |  |  |  |  |  |  |
| Rural | — | — |  |  |  |  |  |  | — | — |  |  |  |  |  |  | — | — |  |  |  |  |  |  | — | — |  |  |  |  |  |
| Urban | 0.75 | 0.56-1.00 | 0.052 |  |  |  |  |  | 0.66 | 0.40-1.10 | 0.110 |  |  |  |  |  | 0.70 | 0.46-1.08 | 0.110 |  |  |  |  |  | 1.04 | 0.50-2.20 | 0.910 |  |  |  |  |
| **Tumor Features** |  |  |  |  |  |  |  |  |  |  |  |  |  |  |  |  |  |  |  |  |  |  |  |  |  |  |  |  |  |  |  |
| **Tumor Site** |  |  |  |  |  |  |  |  |  |  |  |  |  |  |  |  |  |  |  |  |  |  |  |  |  |  |  |  |  |  |  |
| Supratentorial | — | — |  |  |  |  |  |  | — | — |  |  |  |  |  |  | — | — |  |  |  |  |  |  | — | — |  |  | — | — |  |
| Non-supratentorial | 1.24 | 0.78-1.99 | 0.370 |  |  |  |  |  | 1.74 | 0.84-3.60 | 0.130 |  |  |  |  |  | 0.69 | 0.32-1.48 | 0.340 |  |  |  |  |  | 2.63 | 0.80-8.73 | 0.110 |  |  |  |  |
| Unknown |  |  |  |  |  |  |  |  |  |  |  |  |  |  |  |  |  |  |  |  |  |  |  |  |  |  |  |  |  |  |  |
| **Laterality** |  |  |  |  |  |  |  |  |  |  |  |  |  |  |  |  |  |  |  |  |  |  |  |  |  |  |  |  |  |  |  |
| Non-bilateral | — | — |  |  |  |  |  |  | — | — |  |  |  |  |  |  | — | — |  |  |  |  |  |  | — | — |  |  |  |  |  |
| Bilateral | 1.34 | 1.06-1.71 | **0.016** |  |  |  |  |  | 1.63 | 1.10-2.41 | **0.014** |  |  |  |  |  | 1.13 | 0.87-1.47 | 0.380 |  |  |  |  |  | 1.18 | 0.53-2.67 | 0.690 |  |  |  |  |
| Unknown |  |  |  |  |  |  |  |  |  |  |  |  |  |  |  |  |  |  |  |  |  |  |  |  |  |  |  |  |  |  |  |
| **No. of in situ/malignant tumors** |  |  |  |  |  |  |  |  |  |  |  |  |  |  |  |  |  |  |  |  |  |  |  |  |  |  |  |  |  |  |  |
| 1 | — | — |  |  | — | — |  |  | — | — |  |  | — | — |  |  | — | — |  |  | — | — |  |  | — | — |  |  |  |  |  |
| >1 | 2.05 | 1.41-2.99 | < **0.001*** |  | — | — | — |  | 2.21 | 1.29-3.80 | **0.004*** |  | — | — | — |  | 2.60 | 1.30-5.18 | **0.008*** |  | — | — | — |  | 0.78 | 0.23-2.65 | 0.690 |  |  |  |  |
| **Primary Lesion** |  |  |  |  |  |  |  |  |  |  |  |  |  |  |  |  |  |  |  |  |  |  |  |  |  |  |  |  |  |  |  |
| Yes | — | — |  |  | — | — |  |  | — | — |  |  | — | — |  |  | — | — |  |  | — | — |  |  | — | — |  |  |  |  |  |
| No | 2.05 | 1.41-2.99 | < **0.001*** |  | 2.12 | 1.44-3.11 | < **0.001** |  | 2.21 | 1.29-3.80 | **0.004*** |  | 2.27 | 1.31-3.92 | **0.003** |  | 2.60 | 1.30-5.18 | **0.008*** |  | 2.63 | 1.31-5.27 | **0.006** |  | 0.78 | 0.23-2.65 | 0.690 |  |  |  |  |
| **Histological Type** |  |  |  |  |  |  |  |  |  |  |  |  |  |  |  |  |  |  |  |  |  |  |  |  |  |  |  |  |  |  |  |
| GBM subtype | — | — |  |  |  |  |  |  | — | — |  |  |  |  |  |  | — | — |  |  |  |  |  |  | — | — |  |  |  |  |  |
| Non GBM subtype | 1.37 | 0.92-2.04 | 0.120 |  |  |  |  |  | 1.10 | 0.64-1.89 | 0.730 |  |  |  |  |  | 1.76 | 1.04-2.98 | **0.034** |  |  |  |  |  | — | — | — |  |  |  |  |
| **Treatment** |  |  |  |  |  |  |  |  |  |  |  |  |  |  |  |  |  |  |  |  |  |  |  |  |  |  |  |  |  |  |  |
| **Surgical Treatment** |  |  |  |  |  |  |  |  |  |  |  |  |  |  |  |  |  |  |  |  |  |  |  |  |  |  | < **0.001*** |  |  |  | < **0.001*** |
| Surgery | — | — |  |  | — | — |  |  | — | — |  |  | — | — |  |  | — | — |  |  | — | — |  |  | — | — |  |  | — | — |  |
| No surgery | 14.90 | 5.19-42.90 | < **0.001*** |  | 17.19 | 5.96-49.59 | < **0.001** |  | 5.37 | 3.19-9.04 | < **0.001*** |  | 6.61 | 3.83-11.41 | < **0.001** |  | — | — | — |  |  |  |  |  | 1.74e^11^ | 0.99-3.06e^11^ |  |  | 1.74e^11^ | 0.99-3.06e^11^ |  |
| Unknown |  |  |  |  |  |  |  |  |  |  |  |  |  |  |  |  |  |  |  |  |  |  |  |  |  |  |  |  |  |  |  |
| **Radiotherapy** |  |  |  |  |  |  |  |  |  |  |  |  |  |  |  |  |  |  |  |  |  |  |  |  |  |  |  |  |  |  |  |
| No | — | — |  |  |  |  |  |  | — | — |  |  |  |  |  |  | — | — |  |  | — | — |  |  | — | — |  |  | — | — |  |
| Yes | 0.94 | 0.64-1.37 | 0.730 |  |  |  |  |  | 1.02 | 0.58-1.79 | 0.940 |  |  |  |  |  | 0.89 | 0.52-1.52 | 0.670 |  |  |  |  |  | — | — | — |  |  |  |  |
| **Chemotherapy** |  |  |  |  |  |  |  |  |  |  |  |  |  |  |  |  |  |  |  |  |  |  |  |  |  |  |  |  |  |  |  |
| No | — | — |  |  |  |  |  |  | — | — |  |  |  |  |  |  | — | — |  |  | — | — |  |  | — | — |  |  | — | — |  |
| Yes | 1.00 | 0.69-1.44 | 0.980 |  |  |  |  |  | 0.98 | 0.57-1.69 | 0.930 |  |  |  |  |  | 1.01 | 0.60-1.71 | 0.960 |  |  |  |  |  | — | — | — |  |  |  |  |

*Covariables with a p-value < 0.01 in the univariate competing risk analysis were added to the multivariable competing risk model.

Boldface type indicates statistical significance with two-sided p < 0.05.

Abbreviation: CI, confidence interval; GBM, glioblastoma; m, month (s); HR, hazard ratio; SEER, Surveillance, Epidemiology, and End-Results; y, year (s)
