# Supplementary material for: Glioblastoma patients’ survival and its relevant risk factors during the pre-COVID-19 and post-COVID-19 pandemic: real-world cohort study in the USA and China
Source: Int J Surg. 2024 Feb 19;110(5):2939–49. doi: 10.1097/JS9.0000000000001224 (PMC11093471; doi:10.1097/JS9.0000000000001224)
Supplement: Supplementary file 9 [file js9-110-2939-s009.docx]

**Supplementary Table 7** Sensitivity analysis of survival-related risk factors during the period from pre- to post-COVID-19 pandemic in both cohorts

| **SEER** | | | | | | | | | | | | | | | | | |
| --- | --- | --- | --- | --- | --- | --- | --- | --- | --- | --- | --- | --- | --- | --- | --- | --- | --- |
| **variables** | **All-cause Mortality** | | | | | | | |  | **GBM-specific Mortality** | | | | | | | |
|  | **2018-2020** | | **2018** | | **2019** | | **2020** | |  | **2018-2020** | | **2018** | | **2019** | | **2020** | |
|  | **aHR (95%CI)** | **p-value** | **aHR (95%CI)** | **p-value** | **aHR (95%CI)** | **p-value** | **aHR (95%CI)** | **p-value** |  | **aHR (95%CI)** | **p-value** | **aHR (95%CI)** | **p-value** | **aHR (95%CI)** | **p-value** | **aHR (95%CI)** | **p-value** |
| **Advanced Age** |  |  |  |  |  |  |  |  |  |  |  |  |  |  |  |  |  |
| PSM | 1.89 (1.79-2.00) | < **0.001** | 1.83 (1.69-1.98) | < **0.001** | 1.85 (1.69-2.02) | < **0.001** | 2.10 (1.83-2.41) | < **0.001** |  | 1.88 (1.77-1.99) | < **0.001** | 1.83 (1.68-1.99) | < **0.001** | 1.86 (1.69-2.04) | < **0.001** | 2.02 (1.75-2.34) | < **0.001** |
| IPTW | 2.05 (1.95-2.16) | < **0.001** | 1.93 (1.79-2.08) | < **0.001** | 2.08 (1.92-2.26) | < **0.001** | 2.31 (2.04-2.61) | < **0.001** |  | 2.02 (1.92-2.13) | < **0.001** | 1.91 (1.77-2.06) | < **0.001** | 2.04 (1.87-2.22) | < **0.001** | 2.28 (2.01-2.60) | < **0.001** |
| **Bilateral Tumor** |  |  |  |  |  |  |  |  |  |  |  |  |  |  |  |  |  |
| PSM | 2.27 (1.77-2.91) | < **0.001** | 1.69 (1.17-2.46) | **0.005** | 2.79 (1.65-4.71) | < **0.001** | 3.58 (1.95-6.56) | < **0.001** |  | 2.43 (1.87-3.14) | < **0.001** | 1.66 (1.14-2.43) | **0.008** | 2.81 (1.68-4.72) | < **0.001** | 3.65 (1.91-7.00) | < **0.001** |
| IPTW | 1.93 (1.65-2.27) | < **0.001** | 1.59 (1.25-2.03) | < **0.001** | 2.06 (1.53-2.77) | < **0.001** | 2.61 (1.91-3.58) | < **0.001** |  | 2.04 (1.74-2.41) | < **0.001** | 1.61 (1.26-2.06) | < **0.001** | 2.22 (1.65-2.98) | < **0.001** | 2.78 (2.01-3.85) | < **0.001** |
| **No Surgery** |  |  |  |  |  |  |  |  |  |  |  |  |  |  |  |  |  |
| PSM | 1.88 (1.67-2.13) | < **0.001** | 1.97 (1.65-2.35) | < **0.001** | 1.79 (1.45-2.19) | < **0.001** | 1.99 (1.42-2.80) | < **0.001** |  | 1.93 (1.71-2.19) | < **0.001** | 2.01 (1.68-2.40) | < **0.001** | 1.71 (1.38-2.12) | < **0.001** | 2.32 (1.69-3.20) | < **0.001** |
| IPTW | 1.57 (1.45-1.70) | < **0.001** | 1.64 (1.46-1.84) | < **0.001** | 1.56 (1.37-1.78) | < **0.001** | 1.44 (1.18-1.74) | < **0.001** |  | 1.60 (1.47-1.73) | < **0.001** | 1.70 (1.51-1.91) | < **0.001** | 1.56 (1.36-1.79) | < **0.001** | 1.53 (1.27-1.83) | < **0.001** |
| **Radiotherapy** |  |  |  |  |  |  |  |  |  |  |  |  |  |  |  |  |  |
| PSM | 0.76 (0.70-0.82) | < **0.001** | 0.81 (0.72-0.91) | < **0.001** | 0.80 (0.70-0.91) | < **0.001** | 0.50 (0.41-0.62) | < **0.001** |  | 0.82 (0.76-0.89) | < **0.001** | 0.81 (0.72-0.91) | < **0.001** | 0.82 (0.72-0.94) | < **0.001** | 0.57 (0.46-0.70) | < **0.001** |
| IPTW | 0.55 (0.52-0.58) | < **0.001** | 0.61 (0.56-0.65) | < **0.001** | 0.60 (0.55-0.65) | < **0.001** | 0.36 (0.32-0.40) | < **0.001** |  | 0.56 (0.54-0.59) | < **0.001** | 0.63 (0.58-0.68) | < **0.001** | 0.61 (0.56-0.66) | < **0.001** | 0.36 (0.32-0.41) | < **0.001** |
| **Chemotherapy** |  |  |  |  |  |  |  |  |  |  |  |  |  |  |  |  |  |
| PSM | 0.35 (0.32-0.38) | < **0.001** | 0.39 (0.35-0.44) | < **0.001** | 0.37 (0.33-0.42) | < **0.001** | 0.21 (0.17-0.25) | < **0.001** |  | 0.35 (0.32-0.37) | < **0.001** | 0.40 (0.35-0.45) | < **0.001** | 0.35 (0.31-0.40) | < **0.001** | 0.20 (0.16-0.24) | < **0.001** |
| IPTW | 0.32 (0.31-0.34) | < **0.001** | 0.38 (0.35-0.41) | < **0.001** | 0.35 (0.32-0.38) | < **0.001** | 0.20 (0.18-0.22) | < **0.001** |  | 0.32 (0.31-0.34) | < **0.001** | 0.38 (0.35-0.42) | < **0.001** | 0.34 (0.32-0.37) | < **0.001** | 0.20 (0.17-0.22) | < **0.001** |
| **CGC** | | | | | | | | | | | | | | | | | |
| **Non-Primary Lesion** |  |  |  |  |  |  |  |  |  |  |  |  |  |  |  |  |  |
| IPTW | 2.03 (1.37-3.02) | < **0.001** | 2.15 (1.21-3.83) | **0.009** | 2.60 (1.33-5.08) | **0.005** | 0.78 (0.23-2.62) | 0.691 |  | 2.06 (1.39-3.07) | < **0.001** | 2.18 (1.23-3.89) | **0.008** | 2.66 (1.360-5.21) | **0.004** | 0.78 (0.23-2.62) | 0.691 |
| **No surgery** |  |  |  |  |  |  |  |  |  |  |  |  |  |  |  |  |  |
| IPTW | 73.14 (7.04-759.61) | < **0.001** | 113.81 (0.05-13.91e^2^) | **0.002** | - | - | - | - |  | 79.30 (7.64-823.23) | < **0.001** | 113.81 (0.05-13.91e^2^) | **0.002** | - | - | - | - |

Boldface type indicates statistical significance with two-sided p < 0.05.

Abbreviation: aHR, adjusted hazard ratio; CGC, Chinese glioblastoma cohort; CI, confidence interval; GBM, glioblastoma; IPTW, inverse probability of treatment weighting; IQR, interquartile range; m, month (s); n, number; PSM, propensity score matching; SEER, Surveillance, Epidemiology, and End-Results; y, year (s)
