# Supplementary material for: Glioblastoma patients’ survival and its relevant risk factors during the pre-COVID-19 and post-COVID-19 pandemic: real-world cohort study in the USA and China
Source: Int J Surg. 2024 Feb 19;110(5):2939–49. doi: 10.1097/JS9.0000000000001224 (PMC11093471; doi:10.1097/JS9.0000000000001224)
Supplement: Supplementary file 10 [file js9-110-2939-s010.docx]

**Supplementary Table 8** Mutual correlations among age, laterality, comprehensive therapy and all-cause mortality in the SEER cohort*

| **Mutual Correlation Analysis 1** | | | |
| --- | --- | --- | --- |
|  | **Comprehensive Therapy** | **Age ≥ 65 years** | **All-cause Mortality** |
| **Comprehensive Therapy** | 1 |  |  |
| **Age ≥ 65 years** | -0.140*** | 1 |  |
| **All-cause Mortality** | 0.245*** | 0.191*** | 1 |
| **Mutual Correlation Analysis 2** | | | |
|  | **Comprehensive Therapy** | **Bilateral Tumor** | **All-cause Mortality** |
| **Comprehensive Therapy** | 1 |  |  |
| **Bilateral Tumor** | -0.078*** | 1 |  |
| **All-cause Mortality** | 0.245*** | 0.037*** | 1 |

*Pearson Chi-square was performed to investigate the correlations among variables. The associations were presented as *r* coefficient in table.

***p < 0.001

Abbreviation: CGC, Chinese glioblastoma cohort; SEER, Surveillance, Epidemiology, and End-Results
