# Supplementary material for: Glioblastoma patients’ survival and its relevant risk factors during the pre-COVID-19 and post-COVID-19 pandemic: real-world cohort study in the USA and China
Source: Int J Surg. 2024 Feb 19;110(5):2939–49. doi: 10.1097/JS9.0000000000001224 (PMC11093471; doi:10.1097/JS9.0000000000001224)
Supplement: Supplementary file 11 [file js9-110-2939-s011.docx]

**Supplementary Table 9** Logistic regression analysis among variables in mediation model

|  | **All-cause Mortality** | |  |  | **Age ≥ 65 years** | |  |  | **All-cause Mortality** | |  |
| --- | --- | --- | --- | --- | --- | --- | --- | --- | --- | --- | --- |
|  | ***β*** | ***t*** | ***p*** |  | ***β*** | ***t*** | ***p*** |  | ***β*** | ***t*** | ***p*** |
| **Comprehensive Therapy** | -0.245 | -23.89 | < **0.001** |  | -0.140 | -13.35 | < **0.001** |  | -0.223 | -21.79 | < **0.001** |
| **Age ≥ 65 years** |  |  |  |  |  |  |  |  | 0.160 | 15.60 | < **0.001** |
| ***R^2^*** | 0.06 | |  |  | 0.02 | |  |  | 0.09 | |  |
| ***F*** | 570.89*** | |  |  | 178.21*** | |  |  | 414.96*** | |  |

Boldface type indicates statistical significance with two-sided p < 0.05.

****P* < 0.001
