# Supplementary material for: Glioblastoma patients’ survival and its relevant risk factors during the pre-COVID-19 and post-COVID-19 pandemic: real-world cohort study in the USA and China
Source: Int J Surg. 2024 Feb 19;110(5):2939–49. doi: 10.1097/JS9.0000000000001224 (PMC11093471; doi:10.1097/JS9.0000000000001224)
Supplement: Supplementary file 12 [file js9-110-2939-s012.docx]

**Supplementary Table 10** Contribution of comprehensive therapy to all-cause mortality mediated by age

|  | **Effect Value** |  | **Bootstrap 95% CI** | |  | **Proportion mediated, %** |
| --- | --- | --- | --- | --- | --- | --- |
|  |  |  | **Lower CI** | **Upper CI** |  |  |
| **Total Effect** | -0.250 |  | -0.271 | -0.230 |  | 9.11 |
| **Direct Effect** | -0.227 |  | -0.248 | -0.207 |  |  |
| **Indirect Effect** | -0.023 |  | -0.027 | -0.018 |  |  |

Abbreviation: CI, confidence interval
