# Supplementary material for: Glioblastoma patients’ survival and its relevant risk factors during the pre-COVID-19 and post-COVID-19 pandemic: real-world cohort study in the USA and China
Source: Int J Surg. 2024 Feb 19;110(5):2939–49. doi: 10.1097/JS9.0000000000001224 (PMC11093471; doi:10.1097/JS9.0000000000001224)
Supplement: Supplementary file 13 [file js9-110-2939-s013.docx]

**Supplementary Table 11** Logistic regression analysis among variables in mediation model

|  | **All-cause Mortality** | |  |  | **Bilateral Lesion** | |  |  | **All-cause Mortality** | |  |
| --- | --- | --- | --- | --- | --- | --- | --- | --- | --- | --- | --- |
|  | ***β*** | ***t*** | ***p*** |  | ***β*** | ***t*** | ***p*** |  | ***β*** | ***t*** | ***p*** |
| **Comprehensive Therapy** | -0.245 | 23.89 | < **0.001** |  | -0.078 | -7.41 | < **0.001** |  | -0.244 | -23.69 | < **0.001** |
| **Bilateral Lesion** |  |  |  |  |  |  |  |  | 0.018 | 1.76 | 0.078 |
| ***R^2^*** | 0.06 | |  |  | 0.01 | |  |  | 0.06 | |  |
| ***F*** | 570.89*** | |  |  | 54.92*** | |  |  | 287.06*** | |  |

Boldface type indicates statistical significance with two-sided p < 0.05.

****P* < 0.001
